# Eyettention II: A dual-sequence architecture for modeling fixation location, within-word landing position, and fixation duration in reading

**DOI:** 10.3758/s13428-026-03118-6

**Published:** 2026-08-03

**Authors:** Shuwen Deng, Cui Ding, David R. Reich, Paul Prasse, Lena A. Jäger

**Affiliations:** 1 Department of Computer Science, University of Potsdam, An der Bahn 2, 14476 Potsdam, Brandenburg Germany; 2 Department of Computational Linguistics, University of Zurich, Zurich, Switzerland; 3 Department of Informatics, University of Zurich, Zurich, Switzerland

**Keywords:** Eye movements, Generative model of eye movements, Eye-tracking, Reading, Psycholinguistics, Dual-sequence model

## Abstract

The way our eyes move while reading provides valuable insights into both the reader’s cognitive processes and the properties of the text. In particular, eye-tracking-while-reading data has been shown to be highly beneficial in various technological applications, such as enhancing and interpreting neural language models and inferring a reader’s characteristics. However, these applications often rely on large-scale, data-driven models, which demand extensive eye-tracking datasets that are challenging to obtain due to the resource-intensive nature of data collection. To address the challenge of data scarcity, we develop Eyettention II, an end-to-end trained deep-learning model capable of generating realistic scanpaths consisting of a complete set of fixation attributes in chronological order, including fixation location, within-word landing position, and fixation duration. Our model is lightweight, efficiently trainable on limited GPU resources, and closely aligned with cognitive theories. We demonstrate that Eyettention II surpasses state-of-the-art models in scanpath prediction and mirrors human-like gaze behavior by capturing key psycholinguistic phenomena. With its robust performance, Eyettention II holds the potential to drive advancements in natural language processing, facilitate piloting the materials of psycholinguistic experiments, and uncover new insights beyond what is explicitly encoded in theoretical cognitive models. Our code and accompanying tutorial are publicly available at https://osf.io/3ucq7/.

## Introduction

The way our eyes move while reading reveals important information about both the reader’s cognitive processes (Rayner, 1998) and the properties of the stimulus text (Rayner, 2009). Over the past decades, extensive research in cognitive psychology (Engbert, Longtin, & Kliegl, 2002; Reichle, Rayner, & Pollatsek, 2003) and psycholinguistics (Engelmann, Vasishth, Engbert, & Kliegl, 2013) has been dedicated to analyzing eye movements during reading and developing computational cognitive models to simulate human reading behavior. These models can be understood as computational implementations of theories about human reading behavior and its underlying cognitive mechanisms. The primary goal of this research line is to deepen our understanding of the cognitive mechanisms involved in reading and, more generally, human language processing. Most existing approaches remain heuristic, rule-based, or hybrid, as cognitive modeling prioritizes interpretability. Consequently, these models typically feature only a small number of free parameters to adhere to Ockham’s razor and to ensure falsifiability. While this enhances transparency, it often constrains their ability to capture complex patterns and adapt to variations across different populations, languages, stimulus layouts, lab setups, and reading tasks.

Recent machine learning research has increasingly leveraged eye-tracking-while-reading for various technological applications. This includes enhancing natural language processing (NLP) models by incorporating eye movement features to enrich text features (Barrett, Bingel, Keller, & Søgaard, 2016; Hollenstein & Zhang, 2019; Mishra, Kanojia, Nagar, Dey, & Bhattacharyya, 2016) or to regularize neural attention mechanisms to make their inductive bias more human-like (Barrett, Bingel, Hollenstein, Rei, & Søgaard, 2018; Sood et al., 2023; Sood, Tannert, Müller, & Bulling, 2020b). Eye movement data has also been employed to gain insights into the interpretability of neural language models (LMs), providing a means to better understand the differences between human and machine language processing and to evaluate the cognitive plausibility of LMs, which underpin state-of-the-art NLP systems (Beinborn & Hollenstein, 2023; Hollenstein, Pirovano, Zhang, Jäger, & Beinborn, 2021b; Hollenstein, Gonzalez-Dios, Beinborn, & Jäger, 2022b; Merkx & Frank, 2021; Sood, Tannert, Frassinelli, Bulling, & Vu, 2020a). Additionally, eye movement data is utilized to infer a reader’s characteristics, such as cognitive conditions like ADHD (Deng et al., 2022) or dyslexia (Haller et al., 2022; Raatikainen et al., 2021), and linguistic skills, including reading comprehension capacity or whether the reader is a native speaker of the stimulus’ language (Ahn, Kelton, Balasubramanian, & Zelinsky, 2020; Berzak, Katz, & Levy, 2018; Reich et al., 2022). For a recent benchmark on predictive modeling from eye movements that includes most of these tasks, see https://eyebench.github.io/ (Shubi et al., 2025).

Data scarcity has implications not only for training models but also for technological applications that rely on real-time gaze input for arbitrary stimuli at deployment. For example, the aforementioned approaches for enhancing LMs with gaze-derived features assume access to eye-movement data at runtime. In principle, such gaze-enhanced models could leverage human gaze patterns to identify relevant passages, for example, when generating a summary of an input text. However, the assumption that gaze data is available for arbitrary input texts is unrealistic for many real-world applications. For example, when a user wants a language model to summarize a given text, it cannot be assumed that eye-movement data for that specific text has been collected beforehand. To overcome this issue, researchers have started using simulated gaze data for enhancing LMs, resulting in advantages across various NLP tasks (Deng, Prasse, Reich, Scheffer, & Jäger, 2023a, 2024; Khurana, Kumar, Hollenstein, Kumar, & Krishnamurthy, 2023; Sood et al., 2020b; Reich, Deng, Björnsdóttir, Jäger, & Hollenstein, 2024).

Finally, one persistent challenge in psycholinguistic experimentation is the limited size of eye-tracking datasets, which has contributed to the replication crisis in the field (Amrhein, Korner-Nievergelt, & Roth, 2017; Collaboration, 2015; Jäger, Mertzen, Van Dyke, & Vasishth, 2020b). Small samples often result in low statistical power, increasing the likelihood of misleading effect estimates and reducing the reliability of research findings (Vasishth, Mertzen, Jäger, & Gelman, 2018). Using simulated data, we can perform statistical power analyses tailored to the specific experimental materials planned for use with human participants. Such simulations provide more realistic expectations of effect sizes and enable more informed and robust study planning. Along the same lines, generative models of eye movements could also be used to pilot the materials for eye-tracking experiments. By simulating readers’ eye-movement patterns in advance, such models could help identify anomalies or potential confounds in the stimuli, prompting revisions to the materials before data collection.

In sum, there is a broad range of potential usages for simulated gaze data:

- In technological applications, including gaze-augmented NLP, simulated eye-tracking data can be employed to (pre-)train complex machine-learning models that require large amounts of training data. For example, simulated eye-tracking data can support the pre-training of models designed to infer a reader’s characteristics (Prasse, Reich, Makowski, Scheffer, & Jäger, 2024). In this approach, a model is first pre-trained on simulated eye-tracking data (or on a mixture of simulated and real data) to learn generalizable eye-movement patterns, and then fine-tuned on a smaller set of real human eye-tracking recordings for a specific task. For instance, a model predicting reading comprehension could be pre-trained on synthetic gaze data in a self-supervised way and subsequently fine-tuned using real gaze recordings paired with readers’ comprehension scores obtained through comprehension questions.
  While current approaches use synthetic data primarily to learn general eye-movement patterns (Prasse et al., 2024), generating scanpaths that are conditioned on specific stimulus characteristics (e.g., text type or layout), on the reader’s goals (e.g., preparing for comprehension questions or summarizing the text), or tailored to specific populations (e.g., L2 readers) or even individual readers could offer additional advantages. Using such targeted synthetic data to augment pre-training or training datasets may further improve model performance. Evidence that increased diversity in training data can be beneficial has already been shown for real eye-tracking data (Reich et al., 2024), suggesting that similarly diverse simulated data could provide additional gains.
- As pointed out above, some modeling approaches – such as gaze-enhanced LMs – use gaze data as additional, auxiliary input. For example, when automatically generating a text summary, eye movements on the input text could help an LM identify the most relevant parts. In such scenarios, however, gaze data is typically not available for the specific text at application time, nor is it necessary that it come from a concrete, real individual. Simulated eye-tracking data can therefore be particularly useful in settings where real-time gaze information is required as input at application time, such as in gaze-augmented NLP.
- Simulated eye-tracking data can be valuable for preparing psycholinguistic experiments, such as piloting stimulus materials or estimating expected effect size on a given set of stimuli, which, in turn, can be used for a statistical power analysis.
- High-performing deep learning models, trained on large-scale data, can capture patterns not explicitly encoded in theoretical frameworks, offering novel insights into factors that influence eye movement behavior, even if such models remain not fully interpretable. For example, they can serve as a tool for investigating which linguistic or visual features are most informative for guiding human reading behavior.

Earlier work on eye movement prediction during reading has predominantly focused on *either* spatial aspects of eye movements, such as fixation location (Bolliger et al., 2023; Deng et al., 2023b) and word skipping probability (Hahn & Keller, 2016; Wang, Zhao, & Ren, 2019), *or* temporal aspects, like aggregated word-level total duration (Hollenstein et al., 2021b; Hollenstein, Chersoni, Jacobs, Oseki, Prévot, & Santus, 2021a, 2022a; Sood et al., 2020b). However, there is a growing need to develop models that can predict complete scanpaths – capturing *both* spatial and temporal dimensions of sequential eye movements during reading – to enable versatile applications across various fields. To this end, we present Eyettention II, a lightweight generative deep-learning model designed to generate human-like scanpaths on text. Eyettention II extends our previous model, Eyettention (Deng et al., 2023b), an attention-based dual-sequence model that established a new state-of-the-art in fixation location prediction. The new model is capable of generating full scanpath attributes, including fixation location, within-word landing position, and fixation duration, significantly broadening its applicability to a wide range of use cases and research questions. In particular, the contribution of our work is as follows:

- We develop an end-to-end trained dual-sequence architecture for scanpath prediction, which can predict comprehensive scanpath attributes, including fixation location, landing position within a word, and fixation duration, achieving state-of-the-art performance. Additionally, we develop a specialized version that can generate scanpaths tailored to specific readers by conditioning the model on reader identity so that it learns and reproduces characteristic reading patterns of individual readers observed during training.
- We present extensive experiments to evaluate our proposed model across a range of application scenarios, both within and across datasets, and for two typologically different languages with different scripts (alphabetic vs logographic).
- We provide qualitative and quantitative inspections of our model’s behavior, including an in-depth analysis of the model’s capability to reproduce key psycholinguistic phenomena observed in human reading behavior, namely effects of lexical frequency, word length, and surprisal, as well as an analysis of the impact of different decoding strategies for generating scanpaths.
- We perform a power analysis using simulated data as a demonstration of how the model can be applied to prepare a psycholinguistic eye-tracking-while-reading study.
- We further investigate which key properties of the LM used in Eyettention as the stimulus encoder influence its ability to predict human reading behavior. In particular, we examine how differences in model architecture (i.e., model family) and the number of parameters affect predictive performance.

## Related work

### Cognitive models of eye movement control in reading

Cognitive models of eye movement control in reading aim to explain when and where readers move their eyes through the interplay of visual perception, text properties, attention, and oculomotor control. Over the past three decades, two models have been particularly influential in cognitive psychology: the E-Z Reader model (Reichle, Pollatsek, Fisher, & Rayner, 1998; Reichle, Rayner, & Pollatsek, 1999; Reichle et al., 2003) and the SWIFT model (Engbert, Nuthmann, Richter, & Kliegl, 2005). Both models account for how word-level variables, such as lexical frequency and predictability, affect fixation durations, word skipping, and regressions. They differ, however, in how attention is allocated across words and in how linguistic processing influences the timing and targeting of saccades during reading.

E-Z Reader and SWIFT can be compared by considering several processing stages: (i) visual and perceptual encoding, (ii) familiarity checking, (iii) saccade timing and target selection, (iv) attention allocation, and (v) post-lexical or sentence-level processing. Both models assume that perceptual input quality decreases with eccentricity. For instance, when a fixated word is long, the following word falls farther into the parafovea, reducing preview benefit due to visual acuity drop-off. However, there are more differences than similarities. E-Z Reader adopts a serial, stage-based account of attention (Posner, Snyder, & Davidson, 1980; Rayner & McConkie, 1976), in which attention is focused on one word at a time, and lexical processing must be completed before attention shifts to the next word. In contrast, SWIFT assumes a gradient of attention distributed across multiple words (Bouma & De Voogd, 1974; Morrison, 1984), allowing parallel lexical processing with the highest processing rate at the fovea and decreasing rates for parafoveal words to the left and right of the fixated word, and extending, though to a lesser extent, to multiple coming words. The models also differ in how linguistic properties influence eye movement control. In E-Z Reader, early-stage familiarity check is modulated by word predictability and frequency. Completing the familiarity check directly triggers saccadic programming, thereby tightly linking progress in linguistic processing to fixation durations. In SWIFT, saccade timing is governed by an internal, autonomous mechanism that is influenced, rather than directly triggered, by linguistic processing (Engbert & Kliegl, 2001), and saccade target selection arises from probabilistic competition across a dynamic activation field. At the post-lexical level, E-Z Reader includes a separate integration stage that can delay or halt eye movements under processing difficulty, whereas SWIFT lacks an explicit integration stage; instead, processing difficulty, functioning like a “brake” (foveal inhibition), slows eye movements by naturally interfering with the autonomous saccade timer.

More recent work extends these classic accounts by integrating eye-movement control with broader theories of language processing. For instance, Engelmann et al. (2013) developed a framework that embeds syntactic processing difficulty into the EMMA eye movement control model (Salvucci, 2001) inside the cognitive architecture ACT-R (Anderson et al., 2004) by linking slow post-lexical processing to regressions, enabling predictions of re-reading behavior based on parsing difficulty. The Über-Reader model (Veldre, Yu, Andrews, & Reichle, 2020) aims to provide a more comprehensive account of reading by embedding eye movement control within an architecture that explicitly models word identification across sentence reading, lexical decision, and word naming tasks, thereby linking eye movements directly to word identification processes. Similarly, SEAM (Rabe, Paape, Mertzen, Vasishth, & Engbert, 2024) combines the SWIFT model with a cue-based memory retrieval framework (Lewis & Vasishth, 2005) to account for how post-lexical processes, such as syntactic dependency completion and similarity-based interference, influence fixation durations and regressive eye movements.

While these models are designed to explain average eye movement phenomena at the group level using cognitively plausible mechanisms, they are typically developed and tested using a single parameter set fit to aggregated data from a small number of participants. Importantly, this does not imply that such models are incapable of capturing individual differences. Indeed, previous work has shown that systematic adjustment of model parameters can account for meaningful individual differences in reading skill, task demands, language experience, and so on (e.g., Mancheva et al., 2015; Reichle et al., 2013). However, in practice, large-scale and systematic modeling of individual differences is relatively uncommon and often takes a secondary role to explaining average effects. Additionally, intentionally using a small number of parameters while ensuring psychological plausibility can limit the models’ ability to generalize to diverse unseen readers or text types. This reduced generalizability limits their effectiveness in broader technological applications.

### Machine-learning models of eye movement control in reading

Early machine-learning models for eye movement control rely on hand-crafted features – explicitly coded, conceptually meaningful variables – extracted from eye-tracking data, such as word length, frequency, and surprisal (Hara, Mochihashi, Kano, & Aizawa, 2012; Matthies & Søgaard, 2013; Nilsson & Nivre, 2009, 2010, 2011). While these features are easily interpretable, a fixed set of hand-crafted features may fail to capture all relevant information and may require manual adjustments to adapt to different reading tasks or datasets. Additionally, the limited number of parameters in these models further constrains their predictive power. More recent approaches have shifted toward neural networks, which exhibit significantly higher predictive performance. Research has focused on predicting aggregated reading measures for each word of the linguistic stimulus, averaged across readers. These measures include word skipping probability (Hahn & Keller, 2016; Wang et al., 2019) and various gaze-related temporal features, including first-pass fixation duration, and total duration (Hollenstein et al., 2021a, b, 2022a; Sood et al., 2020b). This aggregation process, however, can lead to the loss of potentially relevant sequential information, including important aspects of eye movement behavior such as regressions and re-fixations, as well as individual differences in scanpaths. Moreover, related work in scene viewing shows that deep saliency models can help refine mechanistic scanpath models (D’Agostino, Schwetlick, Bethge, & Kuemmerer, 2025), highlighting the importance of modeling full fixation sequences. Recent advancements have addressed this limitation by developing neural networks that predict the complete sequential order of fixations in a scanpath (Bolliger et al., 2023; Deng et al., 2023b; Khurana et al., 2023), significantly broadening the applicability of these models across a wider range of use cases. In this context, a pre-trained LM is integrated as a core component of the proposed architectures to process linguistic information essential for accurate scanpath prediction.

## Eyettention II

All existing generative models of eye movements – both in cognitive psychology and machine learning – share a key conceptual and technical limitation: they model eye movements during reading as a standard (i.e., single-axis) sequence problem. However, one of the key properties of eye-tracking-while-reading data is its dual-sequence nature: The words are ordered following the grammatical rules of the language (*linguistic sequence axis*), whereas the fixations on these words are chronologically ordered (*temporal sequence axis*). As humans do not strictly read from left to right, but rather skip or re-fixate words and regress to previous words, the alignment of the linguistic and the temporal sequence axes poses a major architectural challenge. Neither standard sequence models (i.e., with a single input sequence axis), nor encoder-decoder architectures, which align an input with an output sequence, can handle the *dual-sequence input* structure of eye movements in reading. The Eyettention model (Deng et al., 2023b) addresses this limitation as the first dual-sequence model that simultaneously processes the sequence of words and the chronological sequence of fixations. The alignment between the two sequences is achieved through a cross-sequence attention mechanism. Eyettention II builds upon this framework, extending the original model – which focuses on predicting the fixation location in a scanpath – by also predicting within-word landing positions and fixation durations. These extensions allow Eyettention II to capture both the spatial and temporal dynamics of eye movements during reading, offering a more comprehensive picture of human scanpath patterns.

In brief, Eyettention II is a dual-sequence model that simultaneously processes both the word sequence and the chronological sequence of fixations. It generates scanpaths for a given text stimulus in a causal, autoregressive manner, processing each fixation sequentially. This means that each generated fixation, along with all preceding fixations, is used as input for the neural network to predict the next fixation. Consequently, the prediction of each fixation is based on both the text stimulus and the preceding fixations in the scanpath. We formulate the task of scanpath prediction as a “next fixation prediction” problem, which we detail in the next section. Following this, we introduce the model architecture along with the training procedures.

### Problem formulation

Given a sentence $\textbf{w}=\langle w_1,\dots ,w_m\rangle$ and an initial part $\langle \textbf{f}_1,\dots ,\textbf{f}_{i-1}\rangle$ of the complete scanpath $\langle \textbf{f}_1,\dots ,\textbf{f}_n\rangle$ for the input sentence, the goal is to predict the next fixation $\textbf{f}_i$. Each fixation $\textbf{f}_i = (k_i, l_i, d_i)$ is a vector of a word index $k_i$ with $1\le k_i \le m$, a landing position $l_i$ within the word $w_{k_{i}}$, and a fixation duration $d_i$. For English texts, each $w_{k_{i}}$ represents a word, whereas for Chinese texts, we use $w_{k_{i}}$ to represent characters. The landing position $l_i$ is defined as the normalized horizontal offset in pixel coordinates of a fixation relative to the left boundary of the currently fixed word (for English) or character (for Chinese), yielding values between 0 and 1. This definition offers maximal flexibility to researchers or practitioners using the data generated by Eyettention II, as it is language-independent and allows any desired mapping to linguistically or graphically meaningful units, including letters, morphemes, syllables, or even sub-character units such as Chinese radicals or diacritics in alphabetic languages. We treat the problem of word index prediction as a multi-class classification task, with each class representing a possible word index in a sentence. Specifically, we estimate a likelihood function $P(k_i|\textbf{w},\textbf{f}_1,\dots ,\textbf{f}_{i-1})$ for predicting the word index. In addition, we treat the problems of predicting landing position and fixation duration as regression tasks, producing point estimates for the predicted landing position $\hat{l}_i$ and fixation duration $\hat{d}_i$. This process is iteratively applied for each fixation in the scanpath until either a special end-of-scanpath token is generated or a predefined maximum scanpath length is reached.

### Model architecture

**Fig. 1:**
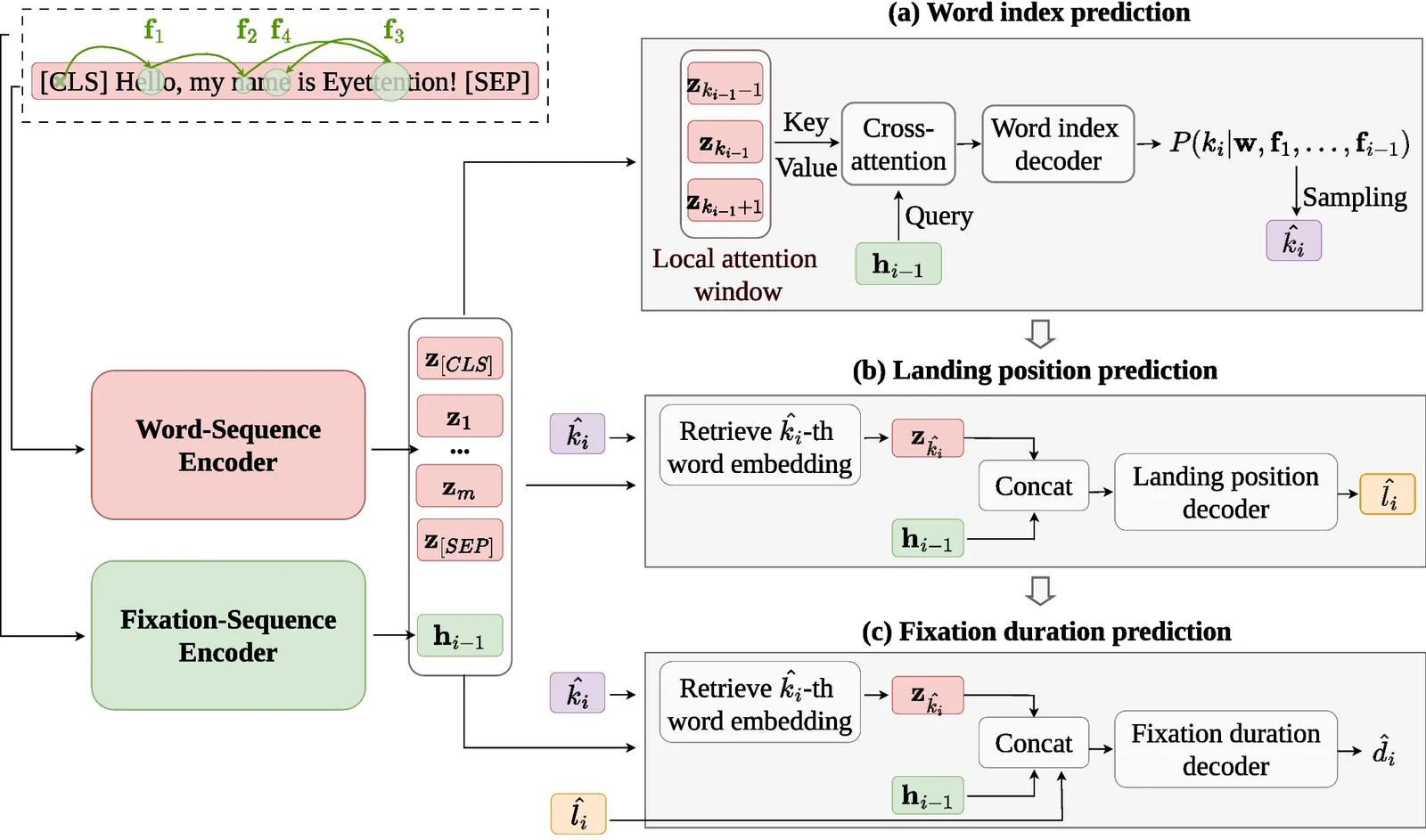
Overview of the Eyettention II model for next fixation prediction. The model predicts three attributes of the next fixation sequentially, with each attribute associated with its own dedicated prediction module: (**a**) Prediction of word index *k*, (**b**) Prediction of landing position *l*, and (**c**) Prediction of fixation duration *d*. The *arrow* → indicates the flow of information between model components, including both inputs and outputs, while the *arrow*  indicates the sequential order in which fixation attributes are predicted

The architecture of Eyettention II closely follows that of the original Eyettention model (Deng et al., 2023b), with two key enhancements: Eyettention II is designed to predict both the spatial and temporal dynamics of scanpaths. This includes not only the word index but also the within-word landing position and the duration of each fixation in a scanpath, expanding beyond the original model’s focus on word index prediction alone. To achieve this, we introduce two additional prediction modules, namely a module for the prediction of within-word landing position described in (b) below and a module for the prediction of fixation duration described in (c), while keeping the other components of the model identical to those of the original Eyettention model. The overall model architecture is depicted in Fig. 1.

#### Overview

The Eyettention II model takes a stimulus sentence or text (a *word sequence*) as input and iteratively generates a sequence of fixations on this stimulus. Each fixation is characterized by its *word index* (the location within the word sequence), its *landing position* within that word, and its *duration*. To generate the next fixation, the model is provided with the initial part of the scanpath, that is, all fixations previously generated for this stimulus. For each fixation, the model not only uses the three fixation attributes (word index, landing position, and fixation duration) but also incorporates semantic information about the fixated word in the form of its word embedding. The first fixation is generated based on a special token that marks the beginning of the scanpath.

A fixation-sequence encoder processes this initial scanpath into a hidden representation, which then “looks at” the stimulus sentence through cross-attention. Crucially, the model attends only to the words around the currently fixated word, thereby mimicking the limited span of the human visual field. The attended word representations are contextualized embeddings computed by the word-sequence encoder, which uses a pretrained language model as its core component.

In sum, the model generates fixations on a given stimulus in an autoregressive manner, that is, it produces them one by one in chronological order, relying only on past fixations, the words that have already been viewed, and the words surrounding the current fixation. In this way, the model simulates human reading behavior, as it does not consult future fixations or words that have not yet been fixated when generating the next fixation.

#### Technical details

In the following, we describe the technical details of all model components. As mentioned above, following (Deng et al., 2023b), Eyettention II employs a dual-sequence bi-encoder structure, where the word-sequence encoder processes a sequence of words $\langle w_1,\dots ,w_m\rangle$ and extracts word embeddings $\langle \textbf{z}_1,\dots ,\textbf{z}_m\rangle$, and the fixation-sequence encoder encodes the initial fixation sequence $\langle \textbf{f}_1,\dots ,\textbf{f}_{i-1}\rangle$ (consisting of the three fixation attributes location (*word index*, *landing position* and *duration*) together with an embedding of the fixated word) into the representation vector $\textbf{h}_{i-1}$ at time i-1. We first describe how the model uses the output of these two encoders to predict the three fixation attributes via the *Word Index Prediction* module (see (a) in Fig. 1), the *Landing Position Prediction* module (see (b) in Fig. 1), and the *Duration Prediction* module (see (c) in Fig. 1). Subsequently, we will describe the technical details of the two encoders.

##### (a) Word index prediction

For predicting a fixation, the model first predicts its location, expressed in terms of the index of the fixated word (*word index*). To this end, the outputs of the two encoders are aligned using a windowed Gaussian version of cross-attention.

Specifically, we use a local attention window $[\textbf{z}_{k_{i-1}-D}, \textbf{z}_{k_{i-1}+D}]$, centered on the embedding of the currently fixated word $\textbf{z}_{k_{i-1}}$, to simulate the human foveal and parafoveal vision, which, in a typical experimental setup, extends a few words to the left and right of the fixation location. We compare the query $\textbf{h}_{i-1}$ with the word embeddings $\textbf{z}_n$ within the window ($k_{i-1}-D\le n \le k_{i-1}+D$), which serve as keys, to compute their attention weights $\textbf{a}_{i}(n)$, reflecting their relevance for deciding where to fixate next. This is achieved using dot-product attention followed by Gaussian smoothing. The Gaussian smoothing is applied to account for the parafoveal information intake around the currently fixated word.

$$\begin{aligned} \textbf{a}_{i}(n) \!=\! \frac{\exp \ \left( \textbf{h}_{i-1}^T W_{a}\textbf{z}_{n}\right) }{\sum _{j=k_{i-1}-D}^{k_{i-1}+D}\exp \ \left( \textbf{h}_{i-1}^T W_{a}\textbf{z}_{j}\right) } \underbrace{\exp \left( \!-\frac{(n-k_{i-1})^{2}}{2\sigma ^{2}}\!\right) }_\text {Gaussian kernel}\ , \end{aligned}$$

where $W_{a}$ represents the learnable weights to align $\textbf{h}_{i-1}$ and $\textbf{z}_n$.

We compute a weighted average of the word embeddings within the window according to their calculated attention weights.

$$ \textbf{c}_{i}=\sum _{n=k_{i-1}-D}^{k_{i-1}+D}\textbf{a}_{i}(n)\textbf{z}_n. $$

We concatenate $\textbf{c}_{i}$ with $\textbf{h}_{i-1}$ to form the final output of the cross-attention mechanism. This combined vector is then fed into the word index decoder, which consists of feed-forward layers with rectified linear unit (ReLU) activation. We consider the saccade target selection as an inherently stochastic process, similar to SWIFT (Seelig et al., 2020) and DeepGaze III (Kümmerer, Bethge, & Wallis, 2022). Consequently, a softmax layer is added as the last layer, which outputs a probability distribution over candidate word indices $P(k_i|\textbf{w},\textbf{f}_1,\dots ,\textbf{f}_{i-1})$.^1^ To compute a point estimate $\hat{k_i}$, we sample from this distribution.

##### (b) Within-word landing position prediction

Eyettention II incorporates a new module specifically for predicting the within-word landing position after determining the next fixated word index $\hat{k_i}$. Once $\hat{k_i}$ is predicted, we retrieve the corresponding word embedding $\textbf{z}_{\hat{k_i}}$ from the output of the word-sequence encoder. This embedding $\textbf{z}_{\hat{k_i}}$ is then concatenated with the fixation sequence representation $\textbf{h}_{i-1}$, and fed into a landing position decoder, which consists of feed-forward layers with ReLU activation. In brief, the next landing position is predicted based on the word to be fixated next and the scanpath history.

##### (c) Fixation duration prediction

Eyettention II includes a new module for predicting fixation duration after determining the next fixated word index $\hat{k_i}$ and landing position $\hat{l_i}$. We concatenate the retrieved word embedding $\textbf{z}_{\hat{k_i}}$, the fixation sequence representation $\textbf{h}_{i-1}$, and the predicted landing position $\hat{l_{i}}$. This combined representation is subsequently fed into a fixation-duration decoder consisting of feed-forward layers with ReLU activations. In brief, the prediction of fixation duration is based on the next word to be fixated, the next landing position, and the scanpath history.

##### Encoders

We build upon the design of the encoders from the original Eyettention model, incorporating a bidirectional word-sequence encoder for embedding the linguistic stimulus sequence and a unidirectional fixation-sequence encoder for embedding the temporal sequence. The bidirectional encoder over the linguistic stimulus enables the model to incorporate both left- and right-contextual information when constructing contextualized token representations. In contrast, the unidirectional Fixation-Sequence Encoder ensures that predictions of future fixations cannot be observed or used, preventing the model from attending to information from fixations that have not yet been generated. Importantly, while the model has access to the entire sentence to build contextualized word representations, it does not have access to future fixations, nor the embeddings of words that have neither been fixated yet nor are contained in the local window around the currently fixated word. This design preserves autoregressivity in the temporal prediction dimension while still enabling the use of rich linguistic information.

**Fig. 2:**
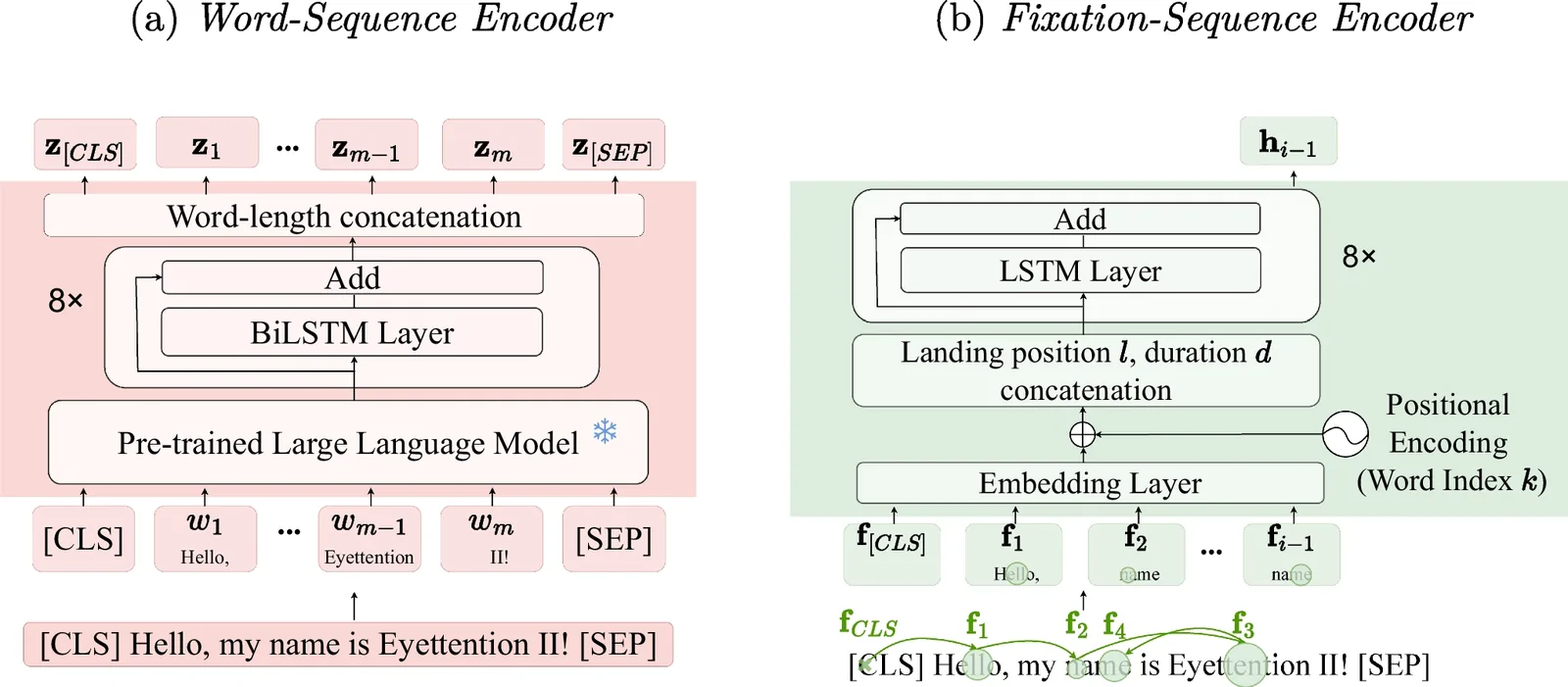
Bi-encoders for processing dual-sequence eye-tracking-while-reading data: (**a**) Word-sequence encoder for embedding the linguistic stimulus, and (**b**) fixation-sequence encoder for embedding the temporal sequence of fixations. The pretrained LLM is frozen during the training of Eyettention II

The word-sequence encoder, depicted in Fig. 2a, transforms a sequence of words into a corresponding list of word embeddings. To achieve this, we employ a pre-trained LLM, such as BERT$_{\text {base}}$ (Devlin, Chang, Lee, & Toutanova, 2019) or RoBERTa$_{\text {base}}$ (Liu et al., 2019), that tokenizes the input sentence into sub-word units and returns their embeddings. The embedding for each word is computed by summing the embeddings of its constituting sub-word tokens. As the LLM remains frozen during the training of Eyettention II, we subsequently feed these word embeddings into a deep stacked bidirectional long short-term memory (BiLSTM) (Graves & Schmidhuber, 2005; Hochreiter & Schmidhuber, 1997) network to encode the relevant linguistic features for the task of scanpath prediction. We newly introduce residual connections among the BiLSTM layers^2^ to facilitate the effective training of the network and enhance model performance. Finally, the output of the BiLSTM network is concatenated with the word length feature to produce the final word embedding $\textbf{z}$.

The fixation-sequence encoder, depicted in Fig. 2b, generates a hidden vector that encodes the context of preceding fixations. We prepend a special “fixation” token $\textbf{f}_0$ for [CLS] to the fixation sequence to denote the start. The input representation for each fixation is constructed by summing the embedding of the fixated word and a trainable position embedding that encodes the word index *k*. The position embeddings are defined as absolute, trainable embeddings learned jointly with the fixation encoder. This follows the standard approach introduced in the Transformer architecture (Vaswani et al., 2017). These input representations are then concatenated with the fixation duration and landing position. To encode the fixation sequence features for the task, we use a deep-stacked LSTM network with residual connections. The output of the fixation-sequence encoder is given by the final hidden state $\textbf{h}_{i-1}$ of the last LSTM layer.

### Reader-specific Eyettention II$_\text {reader}$

Eyettention II is designed to predict generic scanpaths that are not specific to any individual reader. However, eye movements during reading exhibit strong idiosyncratic patterns (Bargary et al., 2017; Jäger et al., 2020a; Makowski et al., 2021), and depending on the intended application, simulating reader- or group-specific scanpaths can be crucial – for instance, for systems tailored to a particular user or for piloting psycholinguistic experiments involving different populations such as L1 and L2 readers. To address this need, we develop Eyettention II$_\text {reader}$, a variant of Eyettention II that simulates the eye-movement behavior of a specific reader.

For training Eyettention II$_\text {reader}$, we are given a set of sentences with associated scanpaths. For each scanpath, the data includes a reader ID, allowing us to build user-specific models. To achieve this, we introduce a trainable reader identifier embedding with an embedding size of $d_{\text {emb}}$ into our model. This embedding is concatenated with the input representation of each fixation in the fixation-sequence encoder.

Importantly, this reader-specific architecture can also be used to simulate group-specific behavior. In practice, this simply requires replacing the reader ID with a group ID. In the computational experiments presented in this paper, we focus on generic and reader-specific scanpaths and leave the modeling of group-specific scanpaths to future work.

### Model optimization

We train the model to generate scanpaths that resemble human eye-movement patterns. To do so, we first need to define a loss function that operationalizes what it means for two scanpaths to “resemble” each other. For simplicity, we define separate loss functions for each of the three fixation attributes and minimize an objective function that combines three loss terms. Specifically, we use negative log-likelihood (NLL) of the predicted *word index* *k*, the mean-squared-error (MSE) between the estimated landing position $\hat{l}$ and the ground-truth landing position *l* in the training data, and the MSE between the estimated fixation duration $\hat{d}$ and the ground-truth fixation duration *d* provided by the training data. The loss function for a scanpath consisting of *I* fixations is defined as:

$$\begin{aligned} \mathcal {L} =&~ \alpha \mathcal {L}_{\text {word index}} + \beta \mathcal {L}_{\text {landing position}} + \gamma \mathcal {L}_{\text {duration}} \\ =&~ \alpha \frac{1}{I}\sum _{i=1}^{I}-logP(k_{i}|\textbf{w},\textbf{f}_{0},\dots ,\textbf{f}_{i-1})\\&+ \beta \frac{1}{I}\sum _{i=1}^{I}\left( \hat{l_{i}} - l_{i}\right) ^{2} + \gamma \frac{1}{I}\sum _{i=1}^{I}\left( \hat{d_{i}} - d_{i}\right) ^{2} \end{aligned}$$

The coefficients α, β, and γ are chosen to balance the three loss terms (see Table 2 for the optimized values after hyperparameter tuning), ensuring they have similar magnitudes in the fully trained model. We use teacher forcing (Williams & Zipser, 1989) instead of auto-regressive training to enable faster and more stable convergence.

## Computational experiments

We evaluate Eyettention II on two typologically different languages with different types of scripts, English and Chinese, using a range of publicly available eye-tracking-while-reading datasets, and compare its performance against state-of-the-art models. Our evaluation includes both within-and across-dataset evaluation to assess the model’s generalization capabilities across various use cases. Lastly, we conduct a series of analyses to gain deeper insights into the model’s effectiveness.

### Datasets

We use four eye-tracking-while-reading corpora to train, tune, and/or evaluate our proposed model and the reference methods. These datasets differ in terms of stimulus language and script (English vs. Chinese), as well as stimulus layout and eye-tracking hardware used for recording. Descriptive statistics for each of the datasets are presented in Table 1.

#### BSC

The *Beijing Sentence Corpus* (BSC) (Pan, Yan, Richter, Shu, & Kliegl, 2021) comprises eye-tracking data collected from native Chinese speakers as they read sentences from Chinese newspapers, with each sentence read by multiple readers.

#### CELER

The *Corpus of Eye Movements in L1 and L2 English Reading* (CELER) (Berzak et al., 2022) contains eye-tracking data from both native (L1) and non-native (L2) English speakers who read English newswire sentences. For the purpose of our study, we use only the data from native speakers (CELER L1). In this dataset, half of the sentences are shared across readers while the other half are unique to each reader.

#### ZuCo

The *Zurich Cognitive Language Processing Corpus* (ZuCo (Hollenstein et al., 2018) and ZuCo 2.0 (Hollenstein, Troendle, Zhang, & Langer, 2020)) contain eye-tracking data from native English speakers who read English movie reviews and Wikipedia articles. These corpora include two distinct reading paradigms: “task-specific” and “natural reading”. We use the “natural reading” subset (ZuCo/ZuCo 2.0 NR) in our study. Each sentence is read by multiple readers.

**Table 1:** Descriptive statistics of the four eye-tracking-while-reading corpora used for model training and evaluation. The number of words per sentence is reported using the mean ± standard deviation

		# Unique	# Words per		
Dataset	Eye-tracker	sentences	sentence	# Readers	Language
BSC	EyeLink II (500 Hz)	150	11.2±1.6	60	Chinese
CELER L1	EyeLink 1000 (1000 Hz)	5456	11.2 ± 3.6	69	English
ZuCo NR	EyeLink 1000 Plus (500 Hz)	700	19.6 ± 9.8	12	English
ZuCo 2.0 NR	EyeLink 1000 Plus (500 Hz)	349	19.6±8.8	18	English

### Reference methods

We include several baseline models in our evaluation to benchmark our model against both established computational cognitive models of eye-movement control in reading and state-of-the-art machine learning approaches.

#### Eyettention (Deng et al., 2023b) & mean baseline

Since the original Eyettention model only predicts fixation locations, but neither landing position within a word nor fixation duration, we benchmark our model against a slightly modified version of Eyettention where we combine the original model with a mean baseline model which predicts landing position and fixation duration as the mean values derived from the training data for these variables.

#### Hollenstein et al. (2021b)

The pre-trained BERT model with a linear dense layer is used to predict fixation duration. The model is fine-tuned on the eye-movement training data.^3^

#### Last (Bestgen, 2021)

This approach feeds a number of hand-crafted features, such as word frequency, word length, POS tags, lexical characteristics, and behavioral measures, into a LightGBM model for fixation duration prediction^3^. It achieved top performance in the 2021 CMCL Shared Task on Eye-Tracking Data Prediction (Hollenstein et al., 2021a).

#### E-Z Reader (Rayner, Li, & Pollatsek, 2007; Reichle et al., 2003)

The E-Z Reader model is a serial, stage-based cognitive model of eye movements in reading, in which attention and linguistic processing are assumed to shift sequentially across words. Lexical processing is divided into two stages: an early familiarity-check stage that triggers saccadic programming, and a later lexical-access stage, after which attention shifts to the next word. The model predicts fixation locations, within-word landing positions, and fixation durations by linking progress in lexical processing to the timing and execution of saccades and by modulating lexical processing with factors such as word frequency and predictability. Higher-level processing difficulty can delay or change planned eye movements, accounting for regressions and increased fixation times under comprehension difficulty.

#### SWIFT (Engbert et al., 2005; Rabe et al., 2021)

The SWIFT model is a cognitive model of eye movement control in reading that proposes parallel processing of multiple words. It predicts fixation positions, within-word landing positions, and fixation durations by incorporating linguistic features such as word length and lexical frequency.

#### Human

We compute inter-reader scanpath similarity by randomly selecting scanpaths from two different individuals reading the same sentence and reporting the mean similarity across all pairs of scanpaths.

**Table 2:** Hyper-parameter grid for the Eyettention II model

Hyper-parameter	Search space	Best value
Landing position decoder: # dense layers	{0, 2, 4, 6}	4
Landing position decoder: # dense units	{64, 128, 256, 512, 1024}	128, 128, 128, 128
Fixation duration decoder: # dense layers	{0, 2, 4, 6}	2
Fixation duration decoder: # dense units	{64, 128, 256, 512, 1024}	1024, 64
α	{0.005, 0.01, 0.02, 0.03, 0.05, 0.1}	0.02
β	{0.1, 0.2, 0.3, 0.4, 0.5, 0.7, 1}	0.3
γ	{0.1, 0.5, 0.8, 1, 3, 5}	1
σ	{0.5, 0.75, 1, 1.25, 1.5, 2, 3}	0.75

**Fig. 3:**
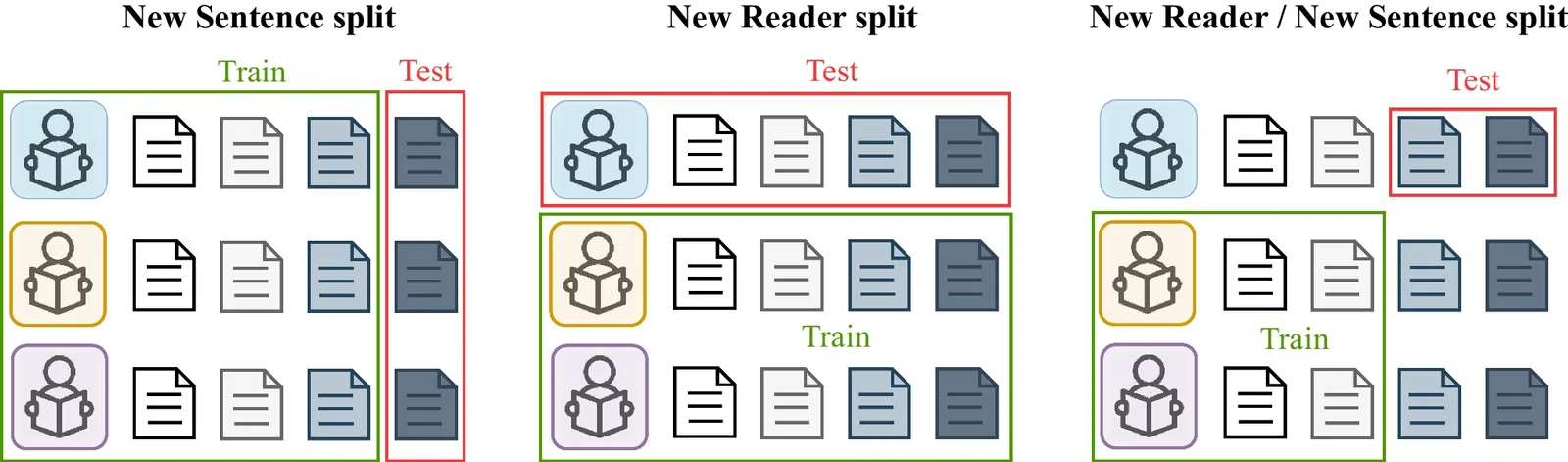
Overview of the three different train/test splits applied in the within-dataset evaluation. *New Sentence split*: Models are tested on scanpaths on sentences not included in the training data, but generated by known readers from whom scanpaths on other sentences are included in the training data. *New Reader split*: Models are tested on scanpaths from new readers who are not included in the training data, on sentences that are included in the training data but read by different readers. *New Sentence / New Reader split*: Models are tested on scanpaths generated by new readers not contained in the training data reading new sentences not contained in the training data either

### Evaluation metrics

There is no established metric for evaluating generative models of eye movements. The different metrics highlight different aspects of scanpath generation. Hence, we use two types of evaluation metrics to assess the models’ performance from different perspectives.

#### Evaluating conditional fixation predictions

We follow the work of Deng et al. (2023b); Kümmerer et al. (2022) to assess each fixation in a scanpath individually, evaluating the model’s ability to predict each fixation when given information about the reader’s previous fixations in a scanpath. This approach examines whether the model and a human reader make similar fixation decisions under identical conditions – that is, when conditioned on the same sequence of prior fixations made by the human reader. Importantly, this evaluation method is parameter-free. We evaluate each fixation attribute separately: negative log-likelihood (NLL) for word index prediction and mean absolute error (MAE) for landing position and fixation duration. MAE for fixation duration is reported in milliseconds (ms), and MAE for landing position is computed on normalized values in [0, 1], where landing position is defined as the horizontal offset (relative to the left boundary of the current word/character) normalized by the word/character width (see Problem Formulation). The NLL measures the average negative log-probability that the model assigns to the true fixation locations in the scanpath observed in the human training data, reflecting how accurately the model predicts each fixation. Lower NLL values indicate that the model assigns higher probabilities to the correct fixation targets, corresponding to better predictive performance. The MAE measures the average absolute deviation between the predicted and observed fixation attributes, indicating how closely the model’s continuous predictions (landing position and fixation duration) align with human data. For each attribute, the final score for a model is computed by first averaging the scores across fixations within each scanpath, followed by averaging across all scanpaths.

#### Evaluating scanpath similarity

We evaluate the quality of the model’s scanpath generation by comparing actual human scanpaths with model-generated scanpaths. For this, we employ the widely adopted MultiMatch metric (Jarodzka, Holmqvist, & Nyström, 2010), which assesses scanpath similarity by computing distances across five dimensions: vector shape, vector length, fixation position, and fixation duration. In this context, “position” and “duration” refer specifically to the components defined within the MultiMatch framework for scanpath-level comparison. These should be distinguished from fixation-level attributes or word-level reading measures used elsewhere in this paper. We apply the default parameters provided in the respective implementations (Wagner, Halchenko, & Hanke, 2019) for consistency.

### Hyperparameter tuning and implementation details

We perform hyperparameter tuning to optimize the newly added prediction modules for landing position and fixation duration. Hyperparameters are model settings that are chosen before training – such as the number of layers or the size of each layer – and can strongly affect model performance. To select them, we use a random grid search. We train the model on the CELER L1 dataset and validate the model on the held-out dataset ZuCo 2.0 NR. The tuning set is discarded after the hyperparameter optimization and is not used in any further experiments. Table 2 shows the hyperparameter grid used for tuning and indicates the configuration found to be optimal. For the remaining model configuration, we maintain the same settings as the previous version of Eyettention (Deng et al., 2023b), which can be found in Table 11 of the Appendix.

We report results using two LLMs in the text encoder: BERT$_{\text {base}}$ (Devlin et al., 2019) and RoBERTa$_{\text {base}}$ (Liu et al., 2019). For each token, we extract its contextualized representation from the final layer of the full pretrained LLM, which is then used as input to the BiLSTM layer. The fixation-sequence encoder uses absolute, trainable position embeddings to represent the word index of each fixation. These embeddings have the same dimensionality as the LLM embeddings, enabling their element-wise summation. These embeddings are initialized randomly and optimized jointly with the model parameters. The entire Eyettention II model comprises 3.7 million trainable parameters. For scanpath generation, we use nucleus sampling with p=0.7 for the BSC data, and temperature sampling with t=0.04 for the CELER data. A detailed analysis of the effect of different decoding strategies can be found in Fig. 6. The network weights are optimized using the Adam optimizer (Kingma & Ba, 2015) with a learning rate of 1e-3. We train the models for 1000 epochs with early stopping (patience of 20 on a validation set sampled from the training data) and a batch size of 256. All neural networks are trained using the PyTorch (Paszke et al., 2019) library on an NVIDIA A100-SXM4-40GB GPU via the NVIDIA CUDA platform.

**Table 3:** Within-dataset evaluation on the BSC (Chinese) dataset using fixation-level metrics. Models are evaluated with negative log-likelihood (NLL) for word index prediction and mean absolute error (MAE) for fixation duration (ms) and landing position (normalized in [0, 1]). The standard error is indicated as the subscript. The dagger † indicates that a model is significantly worse than the best model. Statistical tests were performed using a two-tailed t-test with a significance level α=0.05

	Word index	Fixation duration	Landing position
Model	NLL↓	MAE↓	MAE↓
New sentence split			
Eyettention (Deng et al., 2023b) & Mean	1.854_0.01_†	66.856_0.28_†	0.235_0.00_†
Hollenstein et al. (2021b)	–	65.311_0.27_†	–
Eyettention II-BERT (Ours)	**1.793**_0.01_	61.429_0.29_	**0.208**_0.00_
Eyettention II-RoBERTa (Ours)	**1.793**_0.01_	**61.384**_0.28_	0.209_0.00_
New reader split			
Eyettention (Deng et al., 2023b) & Mean	1.872_0.01_†	65.798_0.31_†	0.235_0.00_†
Hollenstein et al. (2021b)	–	66.715_0.26_†	–
Eyettention II-BERT (Ours)	**1.804**_0.01_	62.588_0.28_	**0.211**_0.00_
Eyettention II-RoBERTa (Ours)	1.809_0.01_	**62.391**_0.29_	**0.211**_0.00_
New sentence / New reader split			
Eyettention (Deng et al., 2023b) & Mean	1.834_0.02_	67.288_0.62_†	0.237_0.00_†
Hollenstein et al. (2021b)	–	65.563_0.62_†	–
Eyettention II-BERT (Ours)	**1.789**_0.02_	62.621_0.63_	**0.215**_0.00_
Eyettention II-RoBERTa (Ours)	1.793_0.02_	**62.584**_0.64_	0.217_0.00_

Bold entries indicate the best results

### Within-dataset evaluation

In the within-dataset evaluation, we train and test the models on the same dataset, dividing the data into separate training and test subsets to assess the models’ in-domain generalization capabilities. The evaluation is performed on two datasets: BSC (Pan et al., 2021), which uses Chinese stimuli, and CELER (Berzak et al., 2022), which uses English stimuli. We explore three distinct train/test splits, each representing a different use-case scenario, as illustrated in Fig. 3. We apply identical data splits across all models to ensure a fair comparison.

**Table 4:** Within-dataset evaluation on the BSC (Chinese) dataset using MultiMatch scanpath similarity metrics. Performance is reported for vector, length, position, and duration dimensions. The standard error is indicated as the subscript. The dagger † indicates that a model is significantly worse than the best model. Statistical tests were performed using a two-tailed t-test with a significance level of α=0.05

	MultiMatch			
Model	Vector↑	Length↑	Position↑	Duration↑
New sentence split				
Eyettention (Deng et al., 2023b) & Mean	0.990_0.00_†	0.981_0.01_†	**0.868**_0.09_	0.726_0.19_†
E-Z Reader (Rayner et al., 2007)	0.956_0.03_†	0.935_0.04_†	0.855_0.07_†	0.636_0.11_†
Eyettention II-BERT (Ours)	**0.993**_0.00_	**0.987**_0.01_	0.864_0.09_†	**0.743**_0.18_
Eyettention II-RoBERTa (Ours)	**0.993**_0.00_	**0.987**_0.01_	0.857_0.09_†	0.731_0.20_†
Human	0.991_0.01_†	0.984_0.01_†	0.856_0.09_†	0.668_0.20_†
New reader split				
Eyettention (Deng et al., 2023b) & Mean	0.990_0.00_†	0.981_0.01_†	**0.866**_0.09_	0.726_0.18_†
E-Z Reader (Rayner et al., 2007)	0.956_0.03_†	0.935_0.04_†	0.856_0.07_†	0.633_0.11_†
Eyettention II-BERT (Ours)	**0.993**_0.00_	**0.987**_0.01_	0.863_0.09_	0.730_0.19_
Eyettention II-RoBERTa (Ours)	**0.993**_0.00_	**0.987**_0.01_	0.862_0.09_†	**0.733**_0.19_
Human	0.991_0.01_†	0.984_0.01_†	0.853_0.09_†	0.663_0.20_†
New sentence / New reader split				
Eyettention (Deng et al., 2023b) & Mean	0.990_0.00_†	0.982_0.01_†	**0.869**_0.08_	**0.746**_0.16_
E-Z Reader (Rayner et al., 2007)	0.957_0.03_†	0.936_0.04_†	0.858_0.07_†	0.640_0.11_†
Eyettention II-BERT (Ours)	**0.993**_0.00_	**0.987**_0.01_	0.864_0.09_	0.738_0.18_
Eyettention II-RoBERTa (Ours)	**0.993**_0.00_	**0.987**_0.01_	0.860_0.09_†	0.733_0.20_†
Human	0.991_0.01_†	0.984_0.01_†	0.857_0.09_†	0.663_0.19_†

Bold entries indicate the best results

**Table 5:** Within-dataset evaluation on the CELER (English) dataset using fixation-level metrics. Models are evaluated with negative log-likelihood (NLL) for word index prediction and mean absolute error (MAE) for fixation duration (ms) and landing position (normalized in [0, 1]). The standard error is indicated as the subscript. The dagger † indicates that a model is significantly worse than the best model. Statistical tests were performed using a two-tailed *t* test with a significance level of α=0.05

	Word index	Fixation duration	Landing position
Model	NLL↓	MAE↓	MAE↓
New sentence split			
Eyettention (Deng et al., 2023b) & Mean	2.226_0.01_†	66.678_0.40_†	0.241_0.00_†
Hollenstein et al. (2021b)	–	66.124_0.40_†	–
Last (Bestgen, 2021)	–	65.952_0.40_†	–
Eyettention II-BERT (Ours)	2.107_0.01_†	63.200_0.40_	0.200_0.00_
Eyettention II-RoBERTa (Ours)	**2.087**_0.01_	**62.828**_0.41_	**0.199**_0.00_
New reader split			
Eyettention (Deng et al., 2023b) & Mean	2.227_0.01_†	66.841_0.40_†	0.241_0.00_†
Hollenstein et al. (2021b)	–	65.912_0.40_†	–
Last (Bestgen, 2021)	–	66.029_0.40_†	–
Eyettention II-BERT (Ours)	2.099_0.01_	63.621_0.41_	**0.199**_0.00_
Eyettention II-RoBERTa (Ours)	**2.091**_0.01_	**63.182**_0.41_	**0.199**_0.00_
New sentence / New reader split			
Eyettention (Deng et al., 2023b) & Mean	2.198_0.01_†	67.891_0.97_†	0.241_0.00_†
Hollenstein et al. (2021b)	–	68.151_0.98_†	–
Last (Bestgen, 2021)	–	67.413_0.98_	–
Eyettention II-BERT (Ours)	2.073_0.01_	**64.936**_0.99_	0.201_0.00_
Eyettention II-RoBERTa (Ours)	**2.056**_0.01_	65.055_1.00_	**0.200**_0.00_

Bold entries indicate the best results

**Table 6:** Within-dataset evaluation on the CELER (English) dataset using the MultiMatch scanpath similarity metrics. Performance is reported for vector, length, position, and duration dimensions. The standard error is indicated as the subscript. The dagger † indicates that a model is significantly worse than the best model. Statistical tests were performed using a two-tailed t-test with a significance level α=0.05

	MultiMatch			
Model	Vector↑	Length↑	Position↑	Duration↑
New sentence split				
Eyettention (Deng et al., 2023b) & Mean	0.975_0.02_†	0.962_0.03_†	0.853_0.12_†	0.719_0.15_†
SWIFT (Rabe et al., 2021)	0.947_0.03_†	0.918_0.04_†	0.757_0.11_†	0.549_0.27_†
E-Z Reader (Reichle et al., 2003)	0.971_0.02_†	0.956_0.03_†	0.841_0.09_†	0.609_0.15_†
Eyettention II-BERT (Ours)	**0.978**_0.02_	**0.967**_0.02_	0.870_0.11_†	0.764_0.14_
Eyettention II-RoBERTa (Ours)	**0.978**_0.02_	**0.967**_0.02_	**0.873**_0.10_	**0.766**_0.14_
Human	0.973_0.02_†	0.958_0.03_†	0.849_0.09_†	0.674_0.17_†
New reader split				
Eyettention (Deng et al., 2023b) & Mean	0.975_0.02_†	0.963_0.02_†	0.860_0.11_†	0.716_0.15_†
SWIFT (Rabe et al., 2021)	0.947_0.03_†	0.918_0.04_†	0.759_0.11_†	0.552_0.27_†
E-Z Reader (Reichle et al., 2003)	0.971_0.02_†	0.956_0.03_†	0.840_0.09_†	0.609_0.14_†
Eyettention II-BERT (Ours)	**0.979**_0.02_	**0.967**_0.02_	**0.866**_0.12_	**0.760**_0.15_
Eyettention II-RoBERTa (Ours)	0.978_0.02_	**0.967**_0.02_	0.865_0.12_	0.757_0.15_
Human	0.973_0.02_†	0.958_0.03_†	0.850_0.09_†	0.676_0.17_†
New sentence / New reader split				
Eyettention (Deng et al., 2023b) & Mean	0.977_0.02_†	0.963_0.02_†	0.845_0.14_†	0.723_0.15_†
SWIFT (Rabe et al., 2021)	0.949_0.03_†	0.921_0.04_†	0.758_0.11_†	0.554_0.28_†
E-Z Reader (Reichle et al., 2003)	0.973_0.02_†	0.957_0.03_†	0.840_0.10_†	0.612_0.15_†
Eyettention II-BERT (Ours)	**0.980**_0.02_	**0.968**_0.02_	0.873_0.10_	**0.769**_0.13_
Eyettention II-RoBERTa (Ours)	**0.980**_0.02_	**0.968**_0.02_	**0.879**_0.10_	0.762_0.14_
Human	0.974_0.02_†	0.959_0.02_†	0.855_0.09_†	0.686_0.15_†

Bold entries indicate the best results

**Table 7:** New sentence split for the reader-specific Eyettention II$_\text {reader}$-BERT. $d_{emb}$ denotes the size of the reader-specific embedding. MAE is reported for fixation duration (ms) and landing position (normalized in [0, 1]). The standard error is indicated as the subscript. The percentages in brackets indicate the percentage improvement over the model without reader embedding. The dagger † indicates models significantly better or worse than the models without reader embeddings. Statistical tests were performed using a two-tailed t-test with a significance level α=0.05

			Word index	Fixation duration	Landing position
Dataset	Model	$d_{emb}$	NLL↓	MAE↓	MAE↓
BSC	Eyettention II	−	1.793_0.01_	61.429_0.29_	0.208_0.00_
(Chinese)	Eyettention II$_\text {reader}$	16	1.674_0.01_† (6.6%)	58.739_0.28_† (4.4%)	**0.204**_0.00_† (1.9%)
	Eyettention II$_\text {reader}$	32	**1.667**_0.01_† (7.0%)	58.257_0.28_† (5.2%)	**0.204**_0.00_† (1.9%)
	Eyettention II$_\text {reader}$	64	1.677_0.01_† (6.5%)	**57.780**_0.28_† (5.9%)	0.207_0.00_† (0.5%)
	Eyettention II$_\text {reader}$	128	1.683_0.01_† (6.1%)	58.112_0.28_† (5.4%)	0.210_0.00_† (-1.0%)
CELER L1	Eyettention II	−	2.107_0.01_	63.200_0.40_	0.200_0.00_
(English)	Eyettention II$_\text {reader}$	16	1.998_0.01_† (5.2%)	60.894_0.38_† (3.6%)	**0.195**_0.00_† (2.5%)
	Eyettention II$_\text {reader}$	32	**1.989**_0.01_† (5.6%)	60.839_0.37_† (3.7%)	**0.195**_0.00_† (2.5%)
	Eyettention II$_\text {reader}$	64	1.991_0.01_† (5.5%)	**60.578**_0.37_† (4.1%)	0.196_0.00_† (2.0%)
	Eyettention II$_\text {reader}$	128	2.004_0.01_† (4.9%)	60.745_0.38_† (3.9%)	0.196_0.00_† (2.0%)

Bold entries indicate the best results

(I) *New Sentence split*: We evaluate how the models perform when predicting scanpaths of a known sample of readers on novel sentences. We conduct five-fold hold-out cross-validation, where the train/test split is based on sentence IDs. In each fold, we use 80% of the sentences for training, and the remaining 20% are reserved for testing, ensuring that all models are tested on sentences not seen during training.

(II) *New Reader split*: We evaluate the models’ capacity to generalize to novel readers when generating scanpaths for known sentences. The model is trained on scanpaths from a subset of readers and tested on a separate set of readers, ensuring that no eye-movement data from the test readers is seen during training. We perform five-fold cross-validation, with an 80/20% train/test split based on reader IDs, ensuring the models are tested on readers not encountered during training.

(III) *New Sentence / New Reader split*: This split examines the models’ ability to generalize to both novel readers and novel sentences at the same time. To maintain an 80/20% train/test split based on both reader IDs and sentence IDs, we perform random re-sampling, repeated five times. Consequently, the models are tested on sentences and readers that are not seen during training.

**Table 8:** Cross-dataset evaluation. Exploring the possibility of generalizing to completely different datasets. MAE is reported for fixation duration (ms) and landing position (normalized in [0, 1]). The standard error is indicated as the subscript

	Training	Fine-tune	Testing	Word index	Fixation duration	Landing position
Model	Data	Data	Data	NLL↓	MAE↓	MAE↓
Eyettention II	ZuCo NR	–	ZuCo NR	2.474_0.02_	36.441_0.33_	0.219_0.00_
Eyettention II	CELER L1	–	ZuCo NR	3.003_0.03_	75.139_0.46_	0.227_0.00_
Eyettention II	CELER L1	ZuCo NR	ZuCo NR	**2.422**_0.02_	**36.046**_0.31_	** 0.213**_0.00_

Bold entries indicate the best results

**Fig. 4:**
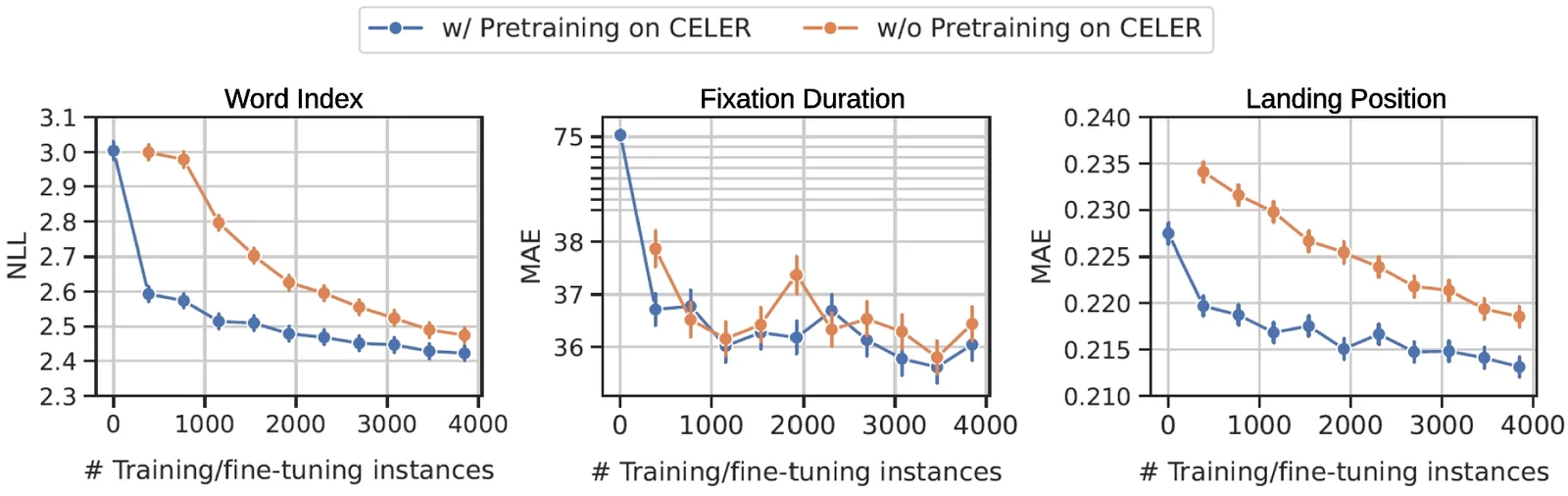
Impact of the amount of data used for training (*orange line*, no pre-training) or fine-tuning (*blue line*, pre-training on CELER) on ZuCo, when testing on held-out data from ZuCo. MAE is reported for fixation duration (ms) and landing position (normalized in [0, 1]). *Error bars* represent the standard error of the mean

#### Results

We present an overview of the results for Eyettention II and the reference methods in Tables 3 and 4 for the BSC (Chinese) dataset, and in Tables 5 and 6 for the CELER (English) dataset. Overall, Eyettention II consistently outperforms all reference methods. Whether BERT or RoBERTa is used in the Word Sequence Encoder, Eyettention II achieves similarly strong performance. In evaluating *conditional fixation prediction* (Tables 3 and 5), Eyettention II improves upon the previous state-of-the-art. In terms of the prediction of fixation location (word index), it outperforms the previous Eyettention model (Deng et al., 2023b), by 2–3% boost on the BSC dataset and by 6% on CELER. In terms of fixation duration prediction, Eyettention II demonstrates a significant advantage over state-of-the-art methods, outperforming (Hollenstein et al., 2021b) and/or Last (Bestgen, 2021) by 5–6% on BSC and 4–5% on CELER. Unlike these methods, which focus on predicting aggregated word-level fixation durations, Eyettention II incorporates scanpath history, leading to superior performance in predicting sequential fixations. For prediction of landing position, Eyettention II surpasses the mean baseline by 9–11% on BSC and 17% on CELER. Furthermore, in evaluating *scanpath similarity* (Tables 4 and 6), Eyettention II significantly outperforms the cognitive models E-Z reader (Rayner et al., 2007; Reichle et al., 2003) and SWIFT (Rabe et al., 2021) across all dimensions of the MultiMatch metrics. The human baseline yields relatively lower similarity scores than our model, reflecting the considerable individual variability in human reading behaviors.

#### Results Eyettention II$_\text {reader}$

We evaluate the performance of Eyettention II$_\text {reader}$ in predicting scanpaths of known individuals on novel sentences by incorporating reader-specific embeddings that encode reader identity. We compare this version to the base Eyettention II model without these identity embeddings, as shown in Table 7. We observe that individualizing the scanpath prediction to specific readers significantly improves the model’s performance across all fixation attributes, highlighting the effectiveness of the Eyettention II$_\text {reader}$ model. The improvements are consistent across various reader embedding sizes ranging from 16 to 128, though using an embedding size of 128 can occasionally lead to worse results.

### Cross-dataset evaluation

In the cross-dataset evaluation, we assess Eyettention II’s ability to generalize to entirely different datasets that it was not exposed to during training. Here, we train the model on the CELER L1 (Berzak et al., 2022) dataset and test it on the ZuCo NR (Hollenstein et al., 2018) dataset. These datasets differ substantially in experimental setup (e.g., eye-tracking hardware, sampling rate, presentation style) and stimulus properties, and were collected in different labs across different countries. Additionally, we explore whether a pretraining/fine-tuning approach could enhance model performance on small datasets, as is often the case in eye-tracking-while-reading applications.

#### Results

In Table 8, we compare the model trained on the CELER dataset and tested on the completely different dataset ZuCo with the model trained and tested both on ZuCo. We use five-fold cross-validation, where 80% of the ZuCo data is used for training and 20% for testing in each fold. We ensure that the same ZuCo test data is used in each fold to evaluate both models for consistency. We observe a significant drop in performance across all three fixation attributes. This indicates the challenge of generalizing to a different dataset when experimental setups and stimulus properties differ substantially. To address this challenge, we explore a *pre-training* and *fine-tuning* strategy where the model is first trained on the CELER dataset, then fine-tuned on 80% of the ZuCo data in each fold, followed by testing on the remaining 20% of ZuCo test data. This strategy yields the best performance, suggesting that the pre-training and fine-tuning strategy can effectively enhance the model’s generalization, especially when working with relatively small eye-tracking datasets. This strategy is particularly valuable for researchers aiming to simulate eye movements for a specific lab setup and stimulus design, but who can only collect a small amount of pilot data due to time or resource constraints. By leveraging available datasets recorded in different labs – even those with differing experimental setups and stimulus properties – researchers can pre-train the model on these external datasets, and then fine-tune it on a small sample recorded in their own target lab. Despite the limited size of the pilot data, our pre-training and fine-tuning strategy enables more accurate and setup-specific eye movement modeling than training on the target data alone.

**Table 9:** Reading measures used for psycholinguistic analyses. We follow the terminology defined by Wilcox, Ding, Sachan, and Jäger (2024); Jakobi, Kern, Reich, Haller, and Jäger (2025) and Oğuz, Ding, Wilcox, & Fuchs (2025)

Reading measures	Description
First fixation duration	Duration of the first fixation on a word.
Gaze duration	The total time spent fixating on a word during the first pass, before the reader either moves forward or makes a regression to an earlier word.
Go past time	Sum of all fixation durations from the first pass on a word until the reader progresses to a word to the right.
Total duration	Total time spent fixating on a word, including re-reading.
First landing position	The relative position within a word where the eye first lands during the initial fixation, normalized by the word’s length (e.g., 0.0 and 1.0 represent the first and last character, respectively, and 0.5 represents the center of the word).
First-pass fixation rate	Proportion of trials in which a word is fixated during the first pass.
First-pass regression rate	Proportion of trials in which a regression is initiated to earlier words during the first pass.
Number of fixations	Total number of fixations on a word.

To further illustrate this, in Fig. 4 we present model performance as a function of the number of training instances from the ZuCo dataset when training it from scratch, compared to the number of fine-tuning instances from ZuCo when the model has been pre-trained on CELER. Notably, with pre-training on CELER, the model achieves significant performance improvements with just a few hundred fine-tuning instances from ZuCo. In contrast, the model trained solely on ZuCo without pre-training requires thousands of instances to reach comparable performance, especially in word index and landing position prediction. This emphasizes the considerable benefits of employing a pre-training approach, particularly in the context of small datasets.

### Investigation of model behavior

#### Psycholinguistic analysis

In this section, we assess Eyettention II’s capability to mirror human-like gaze behavior by investigating whether the model exhibits well-established psycholinguistic phenomena that characterize human reading behavior. Specifically, we will investigate whether the model exhibits effects of *word length, lexical frequency, and surprisal*. From the fixation sequences, we therefore first compute reading measures commonly used in psycholinguistic studies. Definitions of these reading measures are presented in Table 9.

For each reading measure, we then implement a Bayesian (generalized) linear mixed-effects model (Bates et al., 2005; Gelman & Shalizi, 2013; Nicenboim, Schad, & Vasishth, 2025) with this reading measure as target variable and *z*-score normalized *word length, lexical frequency, and surprisal* as predictors. The models are defined as:

1 $$\begin{aligned} \hat{y_{ij}} = {\mathcal {D}}\left( g^{-1}\left( \beta _0 \!+\! b_{0i} \!+\! c_{0j} \!+\! \sum _{m=1}^{M}\left( \beta _m \!+\! b_{mi} \!+\! c_{mj}\!\right) x_m \right) , {\boldsymbol{\theta }_{\mathcal {D}}} \right) \end{aligned}$$

where $y_{ij}$ is the measured response variable and $\hat{y_{ij}}$ the corresponding predicted estimate for participant *i* on item *j*. For continuous reading times, $y_{ij} \in \mathbb {R}$; for fixation and regression rates, $y_{ij} \in \{0, 1\}$; for first landing position, $y_{ij}$ is a value in [0, 1]; and for number of fixations, $y_{ij}$ is a count variable. $\beta _0$ is the global intercept, $b_{0i}$ and $c_{0j}$ represent random intercepts for participants and items, $x_m$ are the M=3 psycholinguistic predictor variables with coefficients $\beta _{m}$ and random slopes for participants $b_{mi}$ and random slopes for items $c_{mj}$.^4^

**Table 10:** Priors for model parameters: $\beta _0$ and $\beta _m$ represent the coefficients of the fixed effects (intercept and psycholinguistic predictors, respectively), *b* denotes random effects, and *sd*(*b*), *sd*(*c*) denote the standard deviation of random effects. Ω is the correlation matrix for random effects; σ is the residual standard deviation for continuous outcomes, and ϕ is the precision parameter for beta-distributed proportions

RTs	First landing pos.	Fix./Regr. rate	Num. fixations
$\beta _0 \sim \textrm{Normal}(6, 1)$	$\beta _0 \sim \textrm{Normal}(0, 1)$	$\beta _0 \sim \textrm{Normal}(0, 1)$	$\beta _0 \sim \textrm{Normal}(0, 1)$
$\beta _m \sim \textrm{Normal}(0, 1)$	$\beta _m \sim \textrm{Normal}(0, 1)$	$\beta _m \sim \textrm{Normal}(0, 1)$	$\beta _m \sim \textrm{Normal}(0, 1)$
$sd(b)\sim \textrm{Exponential}(2)$	$sd(b) \sim \textrm{Exponential}(2)$	$sd(b)\sim \textrm{Exponential}(2)$	$sd(b) \sim \textrm{Exponential}(2)$
$sd(c)\sim \textrm{Exponential}(2)$	$sd(c) \sim \textrm{Exponential}(2)$	$sd(c)\sim \textrm{Exponential}(2)$	$sd(c) \sim \textrm{Exponential}(2)$
$\Omega \sim \textrm{LKJ}(2)$	$\Omega \sim \textrm{LKJ}(2)$	$\Omega \sim \textrm{LKJ}(2)$	$\Omega \sim \textrm{LKJ}(2)$
$\sigma \sim \textrm{Exponential}(2)$	$\phi \sim \textrm{gamma}(2, 1)$	–	–

Focusing on the likelihood family distribution $\mathcal {D}(\cdot )$, continuous reading times are modeled using a lognormal distribution, binary fixation and regression rates using a Bernoulli distribution, first landing position using a beta distribution, and fixation counts using a zero-inflated Poisson distribution. The link function g(·) is the identity function for the lognormal model (on the log scale), the logit function for Bernoulli and beta models, and the log function for the Poisson component of the zero-inflated Poisson model. The vector $\boldsymbol{\theta }_{\mathcal {D}}$ denotes family-specific parameters: a scale parameter σ for the lognormal distribution, a precision parameter ϕ for the beta distribution, no additional parameter for the Bernoulli distribution, and an additional zero-inflation probability for the zero-inflated Poisson model.

**Fig. 5:**
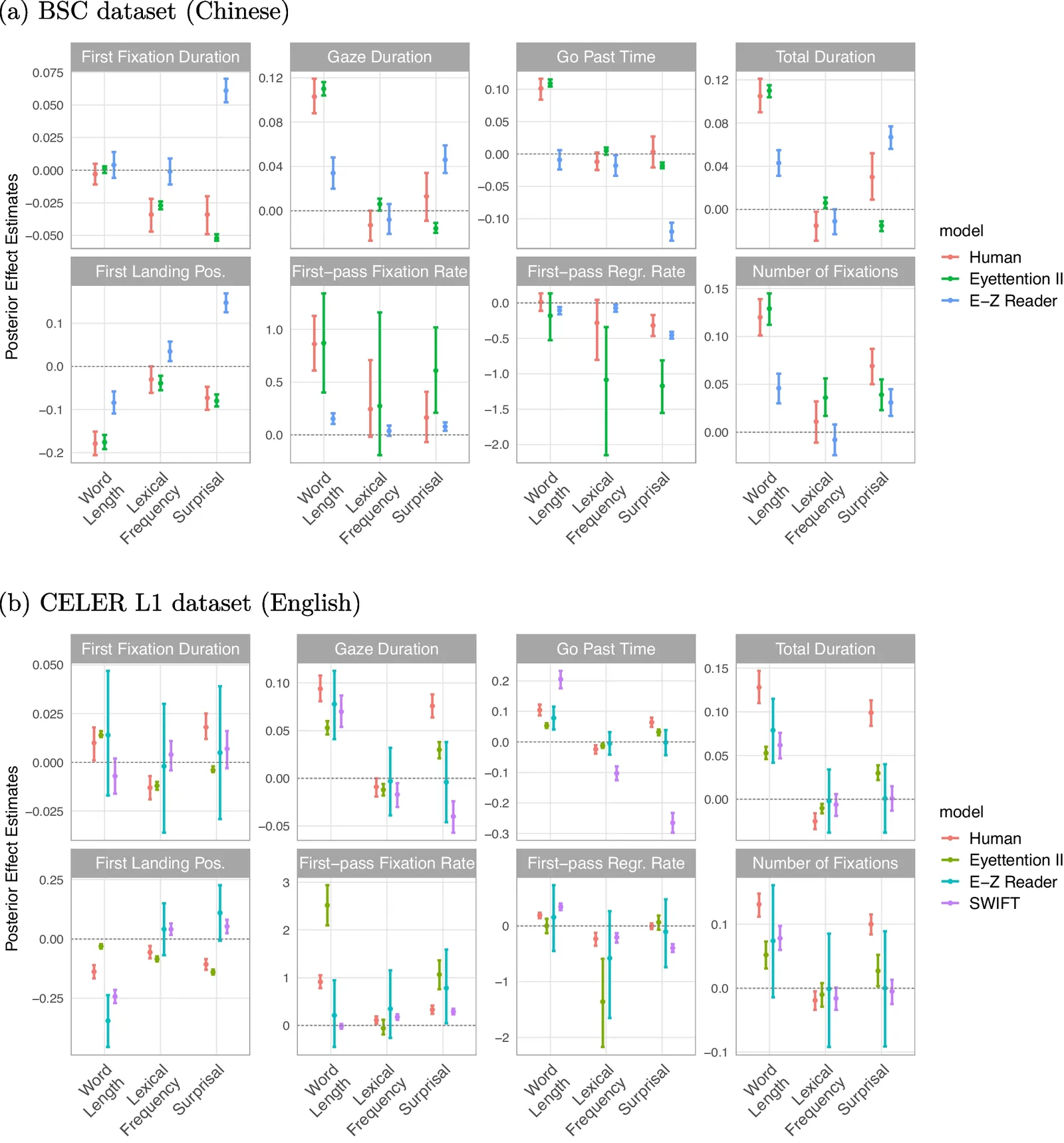
Posterior effect estimates across readers, models, and languages. *Points* show posterior means with 95% credible intervals; *dashed lines* indicate zero. The *y*-axis is on the model scale: log-ms for reading times, log-odds for first landing position and fixation/regression rates, and log-count for number of fixations

The models are fit in *R* (R Core Team, 2024) using the *brms* package (Bürkner, 2021). Each model is fit using four parallel chains of 4000 iterations, with the first 2000 iterations used for warm-up. Mildly informative priors, inspired by Nicenboim et al. (2025), are applied to ensure estimation stability. Details of the priors are provided in Table 10.

##### Results

Figure 5 displays posterior coefficient estimates for *word length, lexical frequency, and surprisal* across eight eye-tracking reading measures from human participants and computational models (Eyettention II, E-Z Reader, SWIFT), separated by language: (a) for the Chinese BSC dataset, and (b) for the English CELER dataset. For all the reading measures, coefficients are interpreted according to their original response scale and the link function g(·) in Eq. 1. For example, in models on reading-time measures, i.e., first fixation duration, gaze duration, go past time, and total duration, the models’ coefficients are on the log-millisecond (log-ms) scale, whereas for first landing position, first-pass fixation rate, and first-pass regression rate, they are on the log-odds scale, and for the number of fixations, log-count scale.

In both the Chinese BSC and English CELER datasets, human reading behavior follows established psycholinguistic effects. Longer words lead to prolonged fixations and more regressions, especially in total duration, gaze duration, and go past time. More frequent words receive shorter fixations and fewer regressions, with the strongest effects in reading time measures. Higher surprisal words increase fixation durations and regressions, particularly in later reading time measures, such as go past time and total duration. First fixation duration and first-pass regression rate show weaker surprisal effects, suggesting surprisal disrupts processing later.

For the computational models, in the Chinese BSC dataset (Fig. 5a), Eyettention II demonstrates strong alignment with human patterns for all three psycholinguistic predictors across most reading measures. However, it struggles with the frequency effect in first-pass regression rate and the surprisal effect in first-pass fixation rate, where its estimates show high uncertainty, reflected in a much wider 95% credible interval. This increased uncertainty may stem from the fact that there are fewer training examples with regressions or skips compared to the reading time measures. The cognitive model E-Z Reader tends to underestimate the word length effect across most measures and fails to capture the surprisal effect in reading time measures.

For the English CELER dataset (Fig. 5b), Eyettention II closely replicates human patterns across most measures. Notably, it outperforms most established cognitive models such as SWIFT and E-Z Reader in most, but not all, measures. While E-Z Reader exhibits greater uncertainty in its estimates (wider 95% credible interval), SWIFT deviates more from human patterns in most of its estimated effect sizes.

Overall, these findings highlight Eyettention II’s strength in aligning with the psycholinguistic phenomena observed in humans across both English and Chinese datasets compared to traditional models. It consistently produced effect size estimates and uncertainty levels that are more closely aligned with human data. For the English dataset, Eyettention II aligns closely with human reading behavior and effectively exhibits phenomena that are key to psycholinguistic theories of human language processing, although – unlike cognitive models – it is not explicitly designed to account for these phenomena. However, the results also suggest areas for improvement, particularly in refining Eyettention II’s performance on binary measures like regression probabilities, to further enhance its generalizability. Its performance on the Chinese dataset could be further refined to match the stronger performance observed in English.

**Fig. 6:**
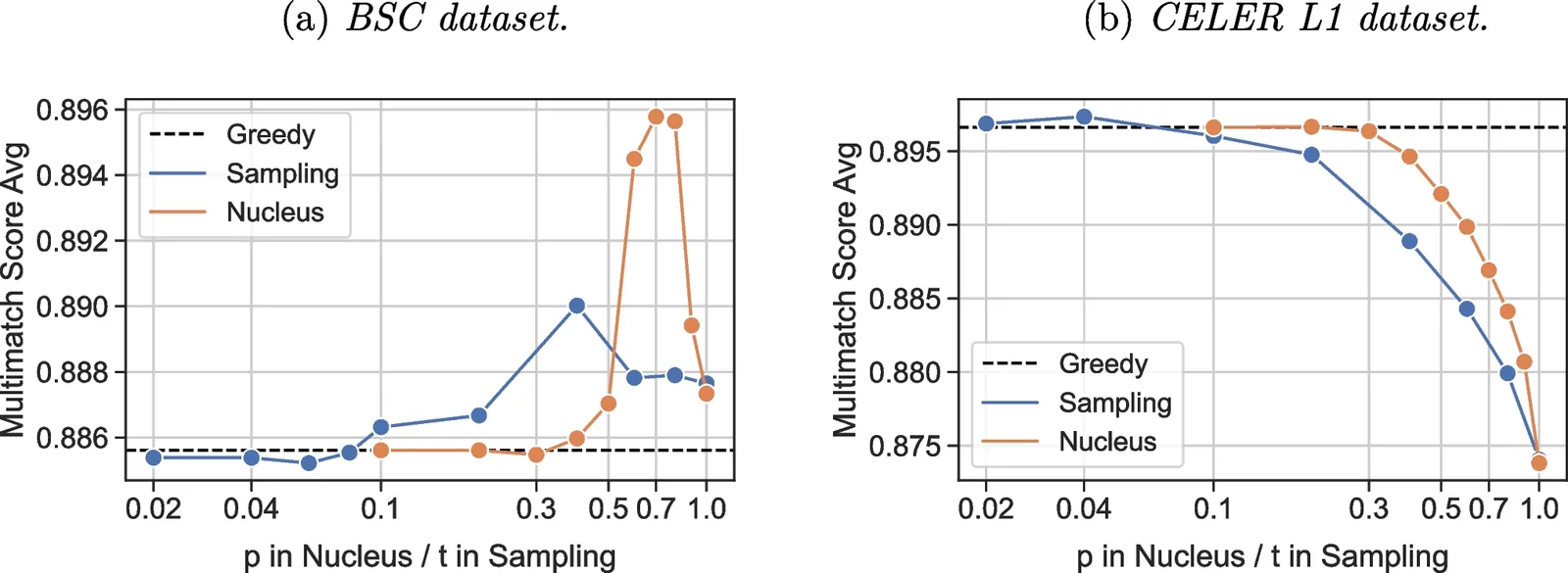
Comparison of scanpaths generated by Eyettention II using different decoding strategies. Higher Multimatch scores indicate greater similarity between the generated scanpaths and observed human scanpaths

#### Decoding strategies for scanpath generation

The Eyettention II model generates fixation locations in a probabilistic manner. Specifically, the word index decoder (see Fig. 1) produces a conditional probability distribution over all possible fixation locations, given the stimulus sentence and the preceding fixations. To determine the next fixation, the model does not simply select the location with the highest probability; instead, it samples from the distribution generated by the word index decoder, resulting in inherently non-deterministic behavior. The procedure used to perform this sampling is referred to as *decoding strategy* and can take different forms, each reflecting a particular trade-off between selecting highly probable fixation locations and allowing for greater variability in the generated scanpaths.

Technically, the word index for each fixation is sampled using a pre-defined decoding strategy. To determine the optimal decoding strategy for Eyettention II, we compare three approaches: (i) *Greedy decoding* selects the next fixated word with the highest likelihood, yielding a deterministic scanpath for a given text. (II) *Temperature sampling* (Ackley, Hinton, & Sejnowski, 1985) adjusts the probability distribution based on a ‘temperature’ hyperparameter *t*. Lower temperatures emphasize high-probability events, producing more likely scanpaths, while higher temperatures introduce variability. (III) *Nucleus sampling* (Holtzman, Buys, Du, Forbes, & Choi, 2020) samples from the smallest set of top-ranked events that cover a cumulative probability threshold *p*.

Results for the BSC dataset are shown in Fig. 6a. Nucleus sampling with p=0.7 achieves the best performance, effectively improving scanpath quality by truncating the less reliable tail of the probability distribution. For the CELER dataset, as shown in Fig. 6b, we observe comparable good performance across the greedy method, low-temperature sampling, and nucleus sampling with small *p* values, with temperature sampling at t=0.04 performing best. Due to the variability in CELER’s training data, which includes longer scanpaths with frequent refixations, regressions, and long-distance saccades, the model learns a more uniform probability distribution for word index prediction. Here, lowering the temperature or nucleus threshold helps reduce randomness, prioritize high-probability events, and ultimately produce high-quality scanpaths.

### Power analysis

We want to ask whether Eyettention II could serve as a reliable tool for experiment planning, particularly in scenarios where human pilot data are scarce or difficult to collect, or no prior literature exists to obtain effect estimates for a certain psycholinguistic manipulation on a given set of stimulus materials. Given its demonstrated ability to mirror human-like gaze behavior – closely imitating the way humans move their eyes while reading – and its alignment with human eye-tracking data with respect to well-established psycholinguistic phenomena, we explore whether Eyettention II can be used to simulate data to conduct a statistical power analysis for a given set of stimulus materials. Specifically, we present results for the New Sentence / New Reader split. This scenario involves conducting a power analysis on new materials, read by a new sample reader that the model has never encountered before. We assume that the lab setups (e.g., eye tracking hardware, sampling rate, presentation style) are similar to the ones used to record the model’s training data.

**Fig. 7:**
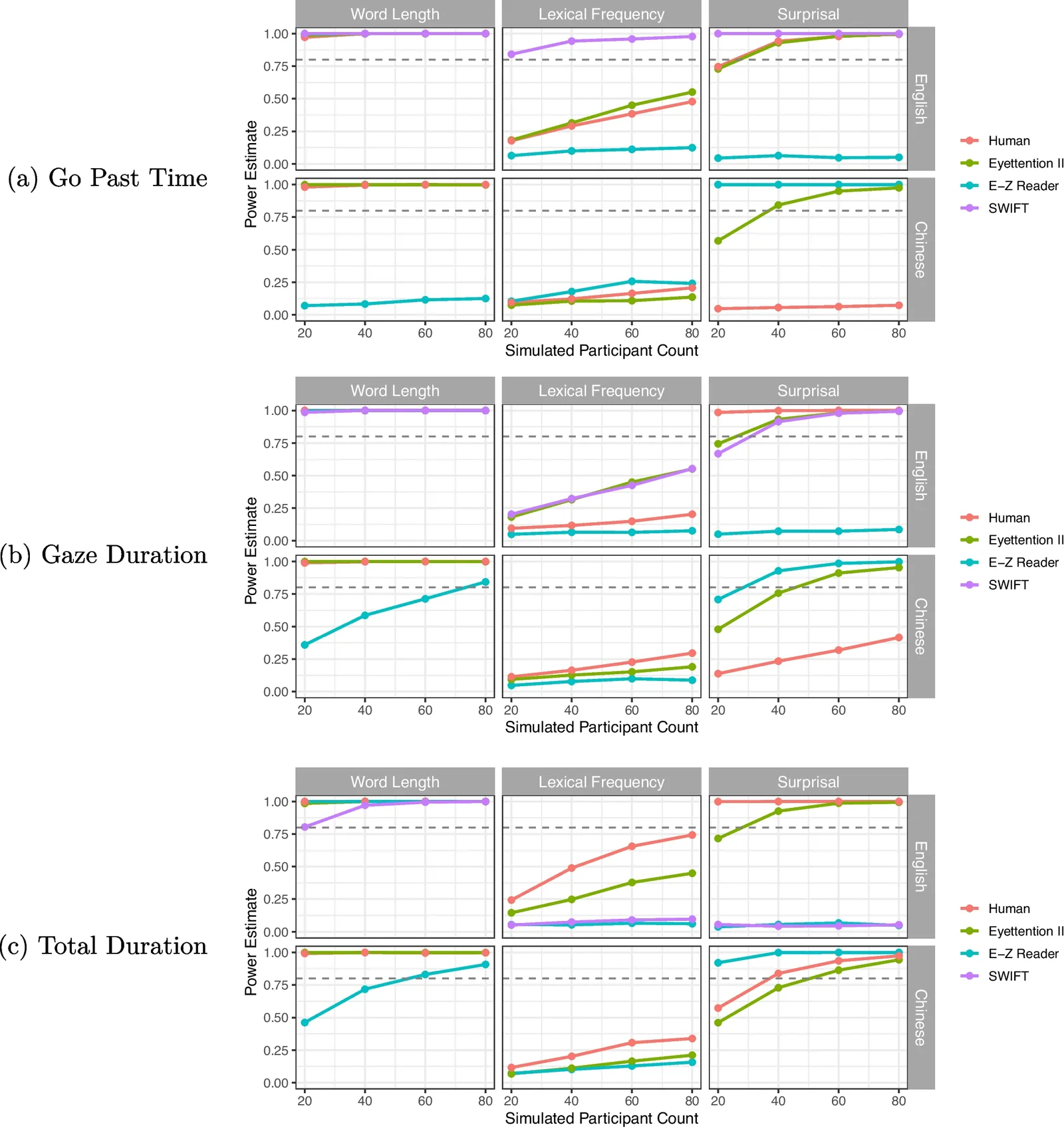
Comparison of simulated power across models and predictors: the predicted statistical power for various participant counts (20, 40, 60, 80) across three predictors: word length, lexical frequency, and surprisal. Results are simulated based on human data and data generated by computational models (Eyettention II, E-Z Reader, and SWIFT) for (**a**) go past time, (**b**) gaze duration, and (**c**) total duration. The *dashed line* indicates a power of 0.8

We conduct an exemplary power analysis for three independent variables of interest: *word length, lexical frequency, and surprisal*, and three dependent variables: *go past time*, *gaze duration*, *total duration*. We compare the power estimates obtained with human data against the results obtained from data simulated with Eyettention II and two reference methods, E-Z Reader and SWIFT, and include data from English CELER and Chinese BSC datasets. For each language, model, and predictor, we simulate 1000 datasets with a fixed item count of 50 and varying participant counts (20, 40, 60, and 80). The simulations are based on parameter estimates (intercepts, predictor effect sizes, and residual standard deviations) obtained from models. Simulated responses are generated using a linear model with fixed effects for predictors and a residual term. A linear regression model is then applied to each simulated dataset, and statistical power is calculated as the proportion of simulations where the p-value for a given predictor is below 0.05. The final power estimates, illustrating the sensitivity of different models to experimental setups and psycholinguistic phenomena, are presented in Fig. 7.

#### Results

Eyettention II closely aligns with human power estimates across all predictors in English, making it an effective tool for power estimation and experimental planning. It follows a power change pattern similar to human data, reaching over 0.8 power with 80 participants for word length and surprisal effects and lower estimated power for lexical frequency effect, between 0.4 and 0.7 for later reading time measures. In contrast, SWIFT consistently overestimates power for lexical frequency and surprisal, reporting near-perfect power even at small sample sizes. Conversely, E-Z Reader underestimates power for these predictors, often below 0.2, regardless of sample size, indicating its limitations in experimental planning.

**Fig. 8:**
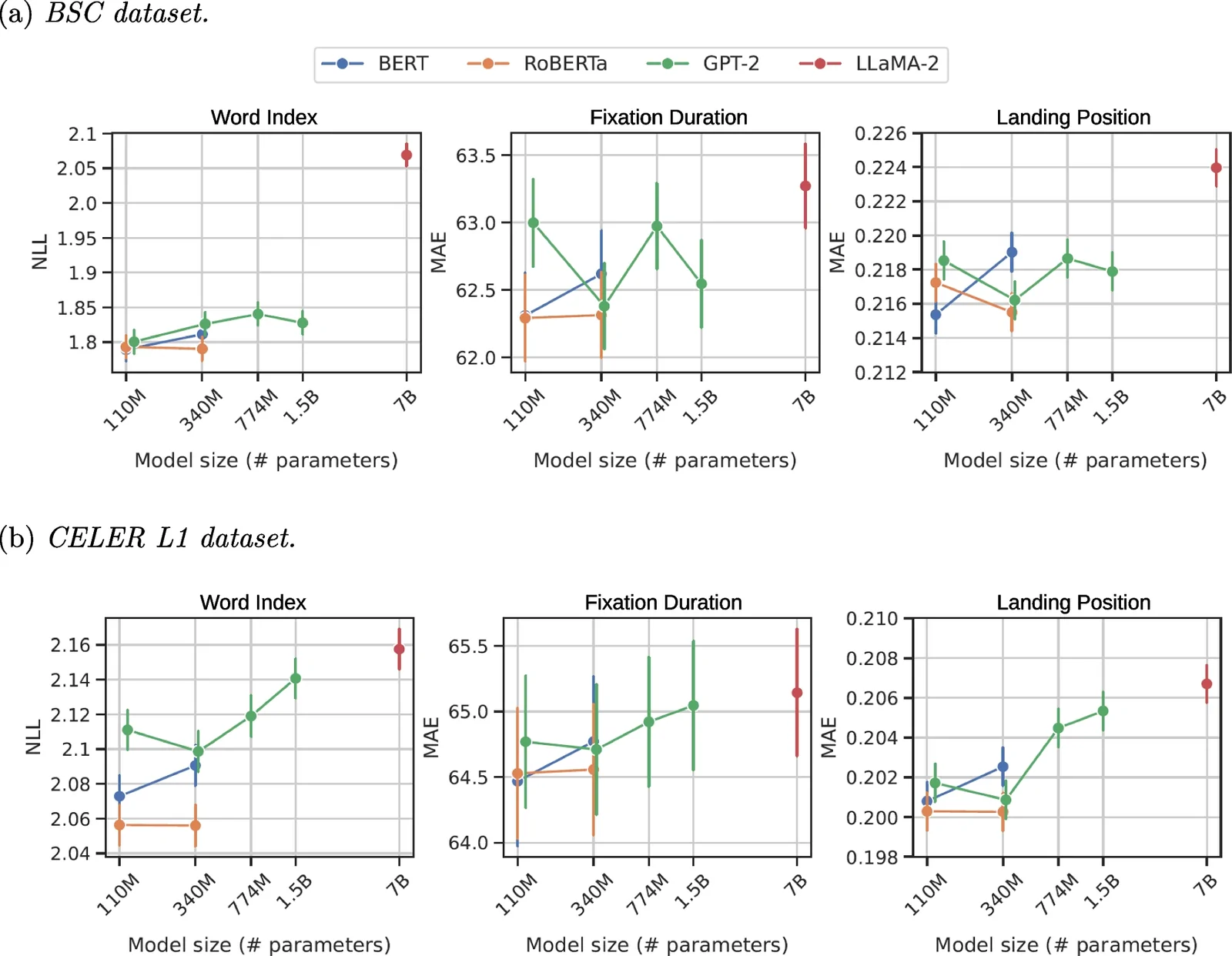
Comparison of different LM families and their variants by size for scanpath prediction. MAE is reported for fixation duration (ms) and landing position (normalized in [0, 1]). *Error bars* represent the standard error of the mean

In the Chinese BSC dataset, Eyettention II closely matches human power estimates for word length and lexical frequency but overestimates power for the surprisal effect in go past time and gaze duration, reaching nearly 1 with 80 participants, whereas human data remain around 0.5. E-Z Reader exhibits even greater overestimation for surprisal, particularly at smaller sample sizes, and it consistently underestimates the power for word length and lexical frequency across the simulated participant counts. These findings indicate that while Eyettention II is highly effective for English experiment planning and outperforms other models for Chinese experiment planning, further refinements are necessary to enhance its accuracy for Chinese psycholinguistic phenomena.

### Inspection of LLMs in scanpath prediction

#### Effects of large-scale LMs on scanpath prediction

Scaling laws for neural LMs suggest that both test loss and downstream task performance improve monotonically with increasing model size (Kaplan et al., 2020). To investigate whether larger-scale LMs provide linguistic features that improve scanpath predictions, we evaluate the performance of Eyettention II with different pre-trained Transformer-based LMs in the word-sequence encoder. This includes two variants of both BERT (Devlin et al., 2019) and RoBERTa (Liu et al., 2019), four variants of GPT-2 (Radford et al., 2019), and LLaMA-2 (Touvron et al., 2023), with the variants differing solely in model size. The size of these models ranges from 110 million to 7 billion parameters. We report results for the New Sentence / New Reader split in Fig. 8.

**Fig. 9:**
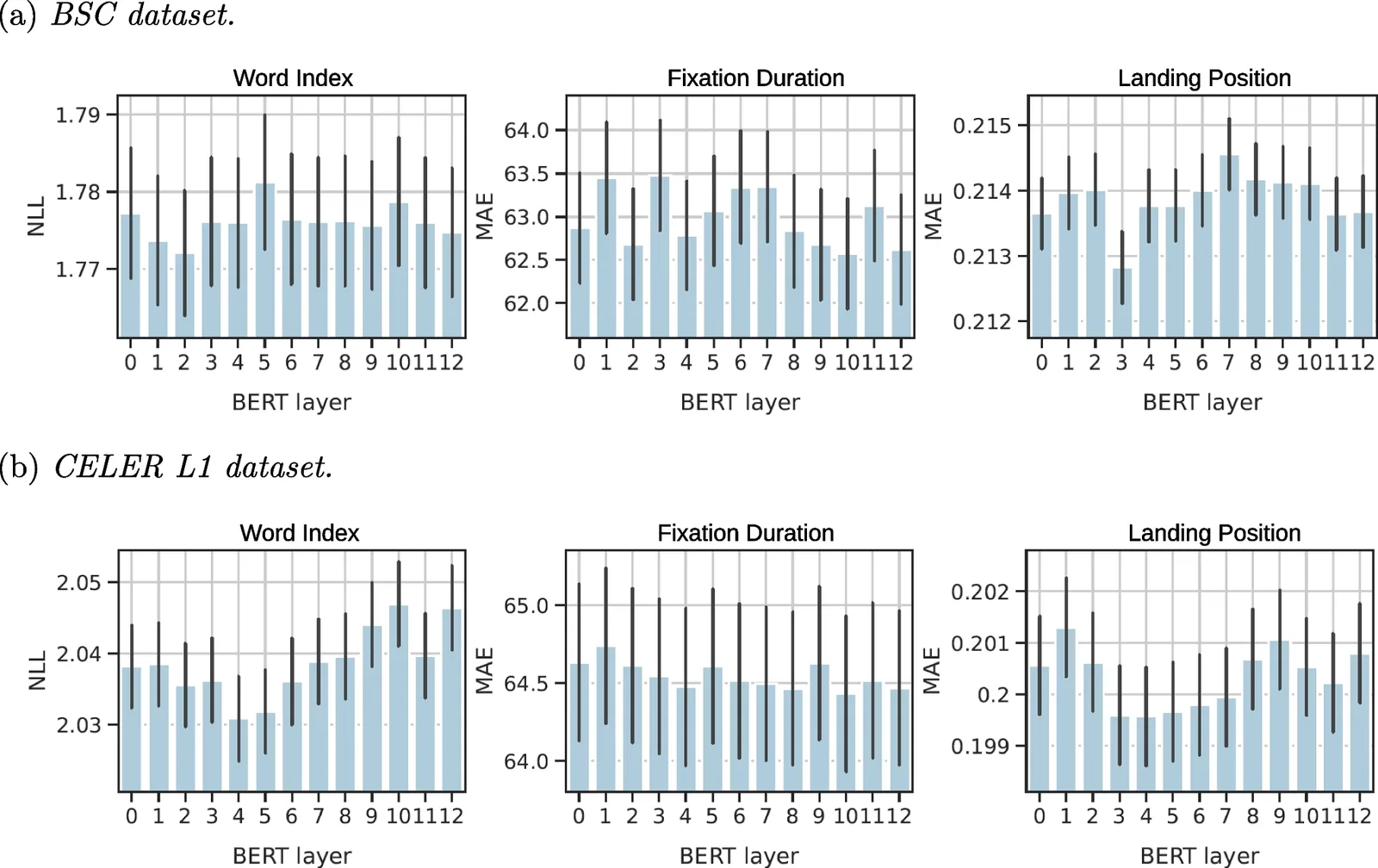
Impact of different BERT layer representations on scanpath prediction. MAE is reported for fixation duration (ms) and landing position (normalized in [0, 1]). *Error bars* represent the standard error of the mean

The main finding is that the smaller variants of LMs within each family, such as BERT 110 million and GPT-2 340 million, demonstrate better performance in predicting human scanpaths compared to their larger counterparts. This suggests that increasing model size does not necessarily improve the ability to model human reading behavior. Additionally, when comparing GPT-2, BERT, and RoBERTa models with similar capacities (110 and 340 million), GPT-2 generally underperforms. This discrepancy may arise from the architectural differences, as BERT and RoBERTa utilize a bidirectional architecture that facilitates the learning of contextual features from both directions, while GPT relies on a unidirectional design.

#### Effect of different levels of LM representations on scanpath prediction

Pre-trained LM embeddings have shown promise in providing linguistic features that support human scanpath prediction during reading. Different layers in these models capture distinct types of linguistic information: BERT’s intermediate layers encode a rich hierarchy of linguistic information, with surface features at the bottom, syntactic features in the middle, and semantic features at the top (Jawahar, Sagot, & Seddah, 2019). We therefore examine how representations from different BERT layers contribute to scanpath prediction. Figure 9 shows the variation in model performance across three fixation attributes for the BSC and CELER datasets. For word index prediction, the lower 2nd layer performs best on BSC, while the 4th layer is most effective on CELER. Fixation duration prediction, however, benefits most from upper layers (10th and 12th) on both datasets. Landing position prediction consistently favors the 3rd layer. These results suggest that phrase-level and surface-level information from lower layers are essential for predicting spatial aspects of scanpaths (word index and landing position), whereas high-level semantic features from upper layers are more influential for temporal aspects (fixation duration). Note that performance differences across layers are small and are likely insufficient to support interpretations in terms of cognitive mechanisms, but rather as indicators of relative predictive utility across representations.

## General discussion

### Transfer learning in scanpath prediction

In cross-dataset evaluations, we explore a pre-training and fine-tuning strategy that demonstrates notable advantages, especially when the target dataset is small. Without pre-training, limited target data alone yields less satisfactory results compared to training on a large amount of data, as shown in Fig. 4, highlighting the challenge of generalizing effectively from small eye-tracking datasets. However, we show that pre-training the model on existing eye-tracking-while-reading data from the same language – despite being collected in different labs with different experimental setups and stimulus properties – enables the model to learn general eye movement patterns. As a result, only a small amount of fine-tuning data recorded under the target setup is needed for adaptation, allowing the model to achieve performance comparable to training from scratch on a large target dataset. Beyond cross-dataset transfer, this strategy has broader applications. For instance, a model pre-trained on natural reading scanpath data could provide a robust starting point for fine-tuning in task-specific scanpath generation, especially with limited target data.

### Larger LMs are less predictive of human reading behavior

An important finding presented in Fig. 8, reveals that the smallest transformer-based LMs, namely BERT and RoBERTa 110 million, generate linguistic features that are superior predictors of both the temporal and spatial aspects of eye movements compared to larger LMs. This observation is consistent with prior research showing that larger transformer-based LMs yield surprisal estimates that are less predictive of human reading times (de Varda & Marelli, 2023; Oh, Clark, & Schuler, 2022; Oh & Schuler, 2023). Our work extends these findings by offering a more comprehensive investigation into how the rich linguistic features (presumably indirectly also encoding predictability estimates) provided by different LMs influence various aspects of eye movement prediction, including both durational (reading times) and spatial (fixation locations) aspects. This contributes to a deeper understanding of the correlation between different LM sizes and the prediction of human reading behavior. The insight that “larger is not better” when modeling human reading behavior implies a need for caution when using pre-trained language models to study human language processing.

### Low-level vs high-level linguistic features in scanpath prediction

While earlier cognitive models of oculomotor control in reading, such as SWIFT and E-Z Reader, focus on low-level text properties, such as word frequency and word length, as key factors influencing scanpath modeling, more recent approaches integrate higher-level factors, such as syntactic processing (Rabe et al., 2024; Veldre et al., 2020). While these cognitive models can be considered computational implementations of theories of reading, machine-learning models are purely data-driven and theory-agnostic. They increasingly use LMs that encode rich linguistic features, showcasing their effectiveness in predicting eye movements during reading. In Fig. 9, we investigate the effectiveness of features extracted from different layers of the BERT model. Lower layers demonstrate greater predictive power for fixation position prediction by effectively capturing phrase-level information and surface features. For example, readers are more likely to skip frequent, shorter words while fixating on longer or syntactically complex phrases as they attempt to parse sentences. On the other hand, fixation duration prediction depends more heavily on higher-level semantic features from upper layers. Readers tend to fixate longer on words or phrases that require greater cognitive effort, especially when semantic ambiguity or complexity is present. Moreover, semantic integration at the sentence or paragraph level can prolong fixation duration as readers try to comprehend the material fully. These findings suggest that both lower-level and higher-level features are crucial for accurately modeling eye movement behavior, with their contributions varying depending on the specific aspect of eye movement being predicted.

### Trade-off between predicting average vs diverse scanpaths

In “Decoding strategies for scanpath generation” section, we investigate different decoding strategies for scanpath generation. Our findings reveal that adjusting the temperature parameter *t* or nucleus threshold *p* enables a flexible trade-off between generating more probable, “average” scanpaths and producing more diverse scanpaths. This adaptability is highly advantageous for different applications. For instance, in psycholinguistic experiments, researchers might prioritize simulating diverse scanpaths to represent variability across readers. Conversely, in NLP tasks, generating average eye movement patterns could be more useful for interpreting language models or for using the eye gaze as feedback in post-training approaches (e.g., when deriving a reward signal from a simulated reader’s eye movement pattern for reinforcement learning with human feedback (RLHF) or related methods). Additionally, we observe that inter-reader similarity scores are lower than the similarity between human data and our model-generated scanpaths. By calibrating the sampling or nucleus parameters, it is possible to align generated scanpath variability with human similarity scores. However, caution is needed to avoid generating incoherent scanpaths if parameters are set too high or too low.

### Bidirectional context in word representations

One limitation of the model’s autoregressive design is that the word embeddings produced by the word-sequence encoder rely on a bidirectional LM and therefore incorporate both left and right context. A more human-like approach would compute each word embedding based only on the words that have already been fixated. However, even this is not necessarily the most cognitively plausible option, as it does not account for shallow processing or for degradation of word representations in memory due to interference or decay. Ongoing work in computational psycholinguistics is investigating which context size and weighting in word embeddings best reflect human processing; see, e.g., Kuribayashi, Oseki, Brassard, and Inui (2022); Mccurdy and Hahn, (2024); Hennert, Paape, Yadav, and Vasishth (2025). In any case, computing cognitively more plausible embeddings would require a different context for every fixation – depending on whether it is a first-pass or later-pass fixation, and including partially masked contexts in cases of skips – which would introduce substantial computational overhead. For simplicity, we therefore use a standard bidirectional model for computing the embeddings. Importantly, the LM in the word-sequence encoder can be replaced with any other type of LM, including a purely autoregressive one, if a different context-conditioning behavior is desired.

### Effects of reduced scanpath variability on power estimation

A potential limitation of the proposed power estimation approach (“Power analysis” section) concerns the variance of model-generated reading measures. Eyettention II tends to produce scanpaths with lower variability than observed in human data, which may lead to overly optimistic power estimates, particularly for predictors such as lexical frequency and surprisal. Since statistical power is highly sensitive to the variance components, even accurate estimates of mean effects can yield inflated power when variability is underestimated. Several strategies could mitigate this limitation in future work. One approach is to calibrate the variance of simulated reading measures using empirical estimates from real eye-tracking datasets, rather than relying solely on model estimates. Furthermore, decoding strategies (“Decoding strategies for scanpath generation” section) that explicitly encourage variability may help address variance underestimation and thereby improve the realism of power predictions.

### Scalability

We develop a lightweight model that can be efficiently trained on limited GPU resources, making it accessible to a broader range of research communities. Its design also minimizes the risk of overfitting to typically small eye movement datasets. The model has the potential to be scaled up, for example by replacing the LSTM layers with self-attention blocks. While such an enhanced model could achieve new state-of-the-art performance, it would require substantial computational resources and more extensive eye-tracking data for training.

## Conclusion

In this work, we introduce Eyettention II, a lightweight scanpath generation model capable of predicting complete fixation attributes – word index, within-word landing position, and fixation duration – in a sequential scanpath given a text stimulus. Additionally, we develop Eyettention II$_\text {reader}$, an extension designed to simulate reader-specific scanpaths. Our experiments demonstrate that Eyettention II achieves state-of-the-art performance and exhibits human-like reading behavior that replicate key statistical and behavioral properties of real human eye movements, making it a versatile tool for diverse applications, such as piloting the materials of psycholinguistic experiments, conducting statistical power analyses that directly take into account the experimental materials of interest, gaze-augmented language modeling to enhance performance on NLP downstream tasks, or pre-training complex machine learning models for applications such as assessments of reading competence and document readability.

## Data Availability

The datasets used in this study were obtained from publicly available sources as shared in the original publications. The BSC dataset (Pan et al., 2021): https://osf.io/vr3k8/. The CELER dataset (Berzak et al., 2022): https://github.com/berzak/celer. The ZuCo dataset (Hollenstein et al., 2018): https://osf.io/q3zws/. The ZuCo 2.0 (Hollenstein et al., 2020) dataset: https://osf.io/2urht/.

## Appendix: Eyettention (Deng et al., 2023b) model configuration

Table 11 presents the model configuration for the previous version of Eyettention (Deng et al., 2023b), which we have kept consistent in our updated model. For the newly integrated modules that predict landing position and fixation duration, see Table 2 for the optimal configuration found during hyperparameter tuning.

**Table 11:** Eyettention (Deng et al., 2023b) model configuration

Parameter	Value
Number of LSTM / BiLSTM layers	8 / 8
LSTM / BiLSTM units	128 / 64
Number of dense layers	4
Number of dense units	512, 256, 256, 256
Embedding dropout rate	0.4
LSTM / BiLSTM dropout rate	0.2
Dense dropout rate	0.2
Attention window size	1

## Footnotes

1 The model’s output is actually a likelihood function over all possible saccade ranges; an intermediate step converts this to the likelihood function over word index by summing the saccade range to the current word index.

2 Due to the differing dimensions between the LLM embeddings and the LSTM hidden states, we have chosen to omit residual connections for the first LSTM layer to avoid introducing many additional parameters by applying linear projections to align the dimensions.

3 To compare with our model, we fit and evaluate the model based on individual fixation durations in a scanpath, rather than aggregated word-level reading measures such as first fixation duration or total duration on a given word as in the original study.

4 By-subject random effects are only included when modeling human data, as participant information is not available for computational models.

## Code Availability

Our code is available at https://osf.io/3ucq7/.
